# Sinensiols H–J, three new lignan derivatives from *Selaginella sinensis* (Desv.) Spring

**DOI:** 10.3762/bjoc.18.146

**Published:** 2022-10-07

**Authors:** Qinfeng Zhu, Beibei Gao, Qian Chen, Tiantian Luo, Guobo Xu, Shanggao Liao

**Affiliations:** 1 School of Pharmacy, Guizhou Medical University, No. 2 Dongqing Road, Guiyang, 550025, P. R. China; 2 State Key Laboratory of Phytochemistry and Plant Resources in West China, Kunming Institute of Botany, Chinese Academy of Sciences, No.132 Lanhei Road, Kunming, 650203, P. R. China

**Keywords:** lignan derivatives, nitric oxide production inhibition, norlignans, *Selaginella sinensis*

## Abstract

One new lignan sinensiol H (**1**) and two new bisnorlignans, sinensiols I and J (**2** and **3**), along with three known compounds were isolated from the whole plants of *Selaginella sinensis*. Their structures were elucidated on the basis of 1D and 2D NMR spectroscopy as well as high-resolution mass spectrometry. The absolute configuration of **1** was established by ECD calculation. Compounds **2** and **3** represent rare examples of naturally occurring 9,9'-bisnorlignans. All the isolated compounds were assayed for their inhibitory effects on LPS-induced nitric oxide production in RAW 264.7 macrophages.

## Introduction

*Selaginella* is the only genus of Selaginellaceae. As a representative of the earliest and still-surviving vascular plant lineage that had arisen about 400 million years ago, it is important for studying the evolution of land plants [1–2]. This genus includes approximately 750 species worldwide, some of which are used in traditional medicines for the treatment of various diseases including diabetes, gastritis, hepatitis, skin diseases and urinary tract infections [3–4]. In fact, *S. tamariscina* and *S. pulvinata* are officially listed in the Chinese Pharmacopoeia for the treatment of amenorrhoea, dysmenorrhoea and traumatic injury [5].

**Figure 1:**
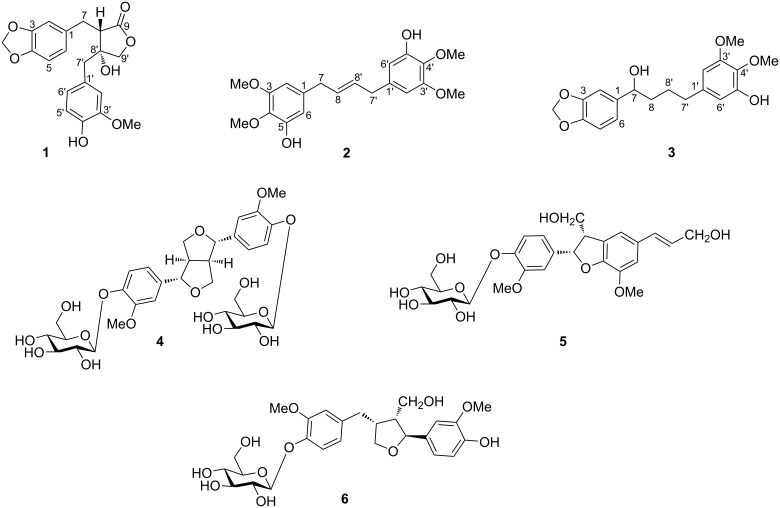
Structures of compounds **1**–**6**.

*Selaginella sinensis,* an endemic species in China, is used as a folk medicine for the treatment of cholecystitis, hepatitis, nephritis, eczema and bleeding [6]. Previous phytochemical studies showed the presence of flavonoids, lignans, glucosides and pigments in the plant [7–8] while pharmacological evaluations showed that some of the compounds possessed anti-oxidant and antiviral activities [9–11]. However, chemical constituents responsible for its efficacy in treating various inflammatory diseases are still not clear. As part of our continuing research on the bioactive compounds from this genus [12–13], the chemical constituents of the whole plant of *S. sinensis* were investigated*.* As a result, three new lignan derivatives **1**–**3** together with three known lignan glycosides **4**–**6** (Figure 1) were isolated. Their isolation, structural elucidation and inhibitory effects on LPS-induced nitric oxide production are reported.

## Results and Discussion

**Figure 2:**
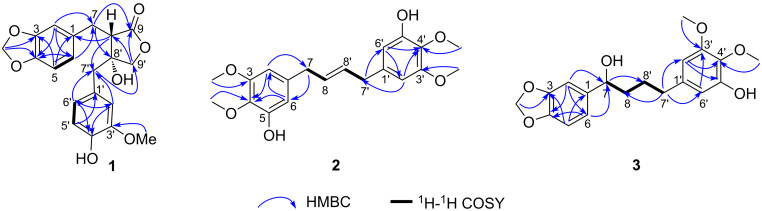
Key HMBC and ^1^H-^1^H COSY correlations of **1**–**3**.

**Figure 3:**
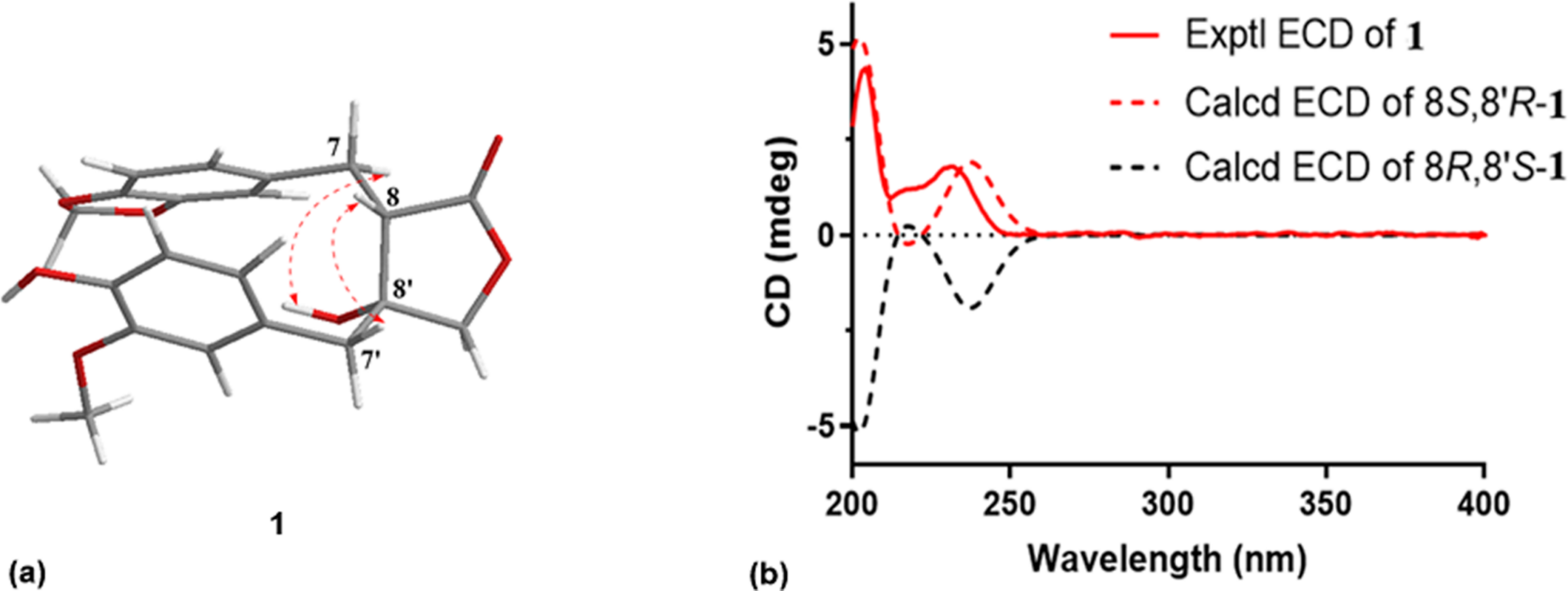
(a) Key ROESY correlations of compound **1**. (b) Experimental and calculated ECD spectra of **1**.

Sinensiol H (**1**) was isolated as a pale yellow amorphous powder. The negative HRESIMS [M − H]^−^ at *m*/*z* 371.1133 (calcd for 371.1136) suggested its molecular formula to be C_20_H_20_O_7_, corresponding to 11 degrees of unsaturation. The IR spectrum showed absorption bands characteristic of hydroxy group (3450 cm^−1^), carbonyl (1765 cm^−1^), and aromatic system (1608, 1516, 1490 cm^−1^). Analysis of its ^1^H NMR (DMSO-*d*_6_) data (Table 1) revealed the presence of two ABX benzene rings [δ_H_ 6.92 (d, *J* = 1.2 Hz, 1H, H-2), 6.83 (d, *J* = 7.9 Hz, 1H, H-5) and 6.79 (dd, *J* = 7.9, 1.2 Hz, 1H, H-6); 6.59 (d, *J* = 1.5 Hz, 1H, H-2′), 6.62 (d, *J* = 8.0 Hz, 1H, H-5′), and 6.47 (dd, *J* = 8.0, 1.5 Hz, 1H, H-6′)]. The ^13^C NMR (Table 1) and HSQC data showed signals due to twelve aromatic carbons, three methylenes (one oxygenated), one oxygenated tertiary carbon, one ester group, one methylenedioxy group (δ_C_ 100.7), one methoxy group (δ_C_ 55.4), and one methine. The chemical shift values of the 1D NMR of **1** were similar to those of the known compound 8′β-hydroxyhinokinin [14], the major difference being the absence of signals for a methylenedioxy (δ_H_ 5.93, δ_C_ 101.2) and the presence of signals for a methoxy group (δ_H_ 3.67, δ_C_ 55.4) in **1**. The HMBC correlations (Figure 2) from 3′-OCH_3_ (δ_H_ 3.67, s, 3H) to C-3′ indicated the methoxy group was located at C-3′. In the ROESY spectrum, the correlations of 8′-OH/H_2_-7 and H-8/H_2_-7′ (Figure 3a) suggested a *trans* orientation of H-8 and 8′-OH. The experimental ECD spectrum of **1** (Figure S16 in Supporting Information File 1) showed two positive Cotton effects (CEs) at 204 and 231 nm, which matched well with those in the calculated ECD curve for the (8S,8′R)-stereoisomer (Figure 3b). Consequently, the structure of **1** was determined as shown in Figure 1, and named sinensiol H.

**Table 1:** ^1^H NMR and ^13^C NMR data of compounds **1**–**3** (δ in ppm and *J* in Hz).

No.	**1** ^a^		**1** ^b^		**2** ^c^		**3** ^c^	

	δ_H_	δ_C_	δ_H_	δ_C_	δ_H_	δ_C_	δ_H_	δ_C_

1		132.9		133.8		138.2		140.6
2	6.85 (d, 1.5, 1H)	109.8	6.92 (d, 1.2, 1H)	109.6	6.34 (d, 1.8, 1H)	105.2	6.84 (s, 1H)	107.4
3		148.0		147.1		154.4		149.1
4		146.4		145.5		135.9		148.1
5	6.76 (d, 7.9, 1H)	108.5	6.83 (d, 7.9, 1H)	108.0		151.4	6.81–6.77 (m, 1H)	108.7
6	6.80 (dd, 7.9, 1.5, 1H)	122.4	6.79 (dd, 7.9, 1.2, 1H)	122.1	6.35 (d, 1.8, 1H)	110.2	6.78–6.74 (m, 1H)	120.5
7	3.13 (dd, 14.5, 5.0, 1H) 2.95 (dd, 14.5, 8.8, 1H)	30.1	2.76–2.71 (m, 2H)	29.1	3.23–3.25 (m, 2H)	39.8	4.54 (t, 6.4, 1H)	74.9
8	2.70 (dd, 8.8, 5.0, 1H)	50.3	2.83–2.78 (m, 1H)	49.9	5.67–5.57 (m, 1H)	131.6	7.82–1.71 (m, 1H) 1.70–1.60 (1H, overlapped)	39.6
9		177.4		178.0				
1′		126.5		127.1		138.2		139.8
2′	6.48 (d, 1.9, 1H)	112.2	6.59 (d, 1.5, 1H)	114.0	6.34 (d, 1.8, 1H)	105.2	6.31 (s, 1H)	105.1
3′		146.9		147.1		154.4		154.3
4′		145.3		145.1		135.9		135.8
5′	6.84 (d, 8.1, 1H)	115.0	6.62 (d, 8.0, 1H)	115.1		151.4		151.2
6′	6.53 (dd, 8.1, 1.9, 1H)	122.7	6.47 (dd, 8.0, 1.5, 1H)	122.4	6.35 (d, 1.8, 1H)	110.2	6.30 (s, 1H)	110.1
7′	2.62 (br s, 2H)	43.1	2.64–2.59 (m, 2H)	41.7	3.23–3.25 (m, 2H)	39.8	2.50 (t, 7.1, 2H)	36.7
8′		78.4		78.0	5.67–5.57 (m, 1H)	131.6	1.70–1.60 (1H, overlapped) 1.60–1.44 (m, 1H)	28.7
9′	4.18 (d, 10.0, 1H) 3.91 (d, 10.0, 1H)	77.0	4.14 (d, 9.4, 1H) 3.81 (d, 9.4, 1H)	75.5				
3-OCH_3_					3.78 (s, 3H)	56.4		
3′-OCH_3_	3.84 (s, 3H)	56.1	3.67 (s, 3H)	55.4	3.78 (s, 3H)	56.4	3.81 (s, 3H)	61.0
4-OCH_3_					3.76 (s, 3H)	61.0		
4′-OCH_3_					3.76 (s, 3H)	61.0	3.76 (s, 3H)	56.3
–OCH_2_O–	5.94 (s, 2H)	101.1	5.96 (s, 2H)	100.7			5.93 (s, 2H)	102.2
4′-OH			8.78 (s, 1H)					
8′-OH			5.38 (s, 1H)					

^a^Recorded at 600/150 MHz for ^1^H/^13^C in CDCl_3_; ^b^recorded at 600/150 MHz for ^1^H/^13^C in DMSO-*d*_6_; ^c^recorded at 600/150 MHz for ^1^H/^13^C in MeOH-*d*_4_.

Compound **2** was obtained as a white amorphous powder. Its molecular formula was determined to be C_20_H_24_O_6_ by the HRESIMS peak at *m*/*z* 359.1497 [M − H]^−^ (calcd for 359.1500). The IR spectrum of **2** showed the presence of hydroxy (3417 cm^−1^) and aromatic (1593, 1509 cm^−1^) groups. The ^1^H NMR spectrum recorded in MeOH-*d*_4_ (Table 1) of compound **2** displayed signals for two aromatic protons at δ_H_ 6.35 (d, *J* = 1.8 Hz, H-6) and δ_H_ 6.34 (d, *J* = 1.8 Hz, H-2), one methine at δ_H_ 5.67–5.57 (m, H-8), one methylene at δ_H_ 3.23–3.25 (2H, m, H_2_-7) and two methyl groups at δ_H_ 3.78 (3H, s, 3-OCH_3_ and δ_H_ 3.76 (3H, s, 4-OCH_3_). The ^13^C NMR spectrum of **2** (Table 1) revealed 10 carbon signals for a benzene, one olefinic carbon, one methylene and two methoxy groups. The above mentioned 1D NMR data of **2** in combination with its molecular formula indicated that the compound must be a symmetrical dimeric benzene derivative. Further analysis of NMR data suggested that the structure of **2** was quite similar to that of (*E*)-5,5′-(but-2-ene-1,4-diyl)bis(3-methoxybenzene-1,2-diol) [15]. The main difference was that the hydroxy group at C-4 and C-4′ in (*E*)-5,5′-(but-2-ene-1,4-diyl)bis(3-methoxybenzene-1,2-diol) was substituted by a methoxy group in **2**, which was confirmed by the HMBC correlation (Figure 2) from δ_H_ 3.76 (4-OCH_3_, 4′-OCH_3_) to δ_C_ 135.9 (C-4, C-4′). The absorption band near 999 cm^−1^ in the IR spectrum (Figure S26 in Supporting Information File 1) indicated that the double bond has an *E* configuration [16–19]. Therefore, the structure of compound **2** was established as shown in Figure 1, and named as sinensiol I.

Sinensiol J (**3**) was isolated as a white amorphous powder. Its HRESIMS showed [M + HCOO]^−^ at *m*/*z* 391.1394 (calcd for 391.1398), consistent with the molecular formula of C_19_H_22_O_6_. The ^1^H and ^13^C NMR data (Table 1) of **3** were extremely similar to those of the *rac*-1-(benzo[*d*][1,3]dioxol-5-yl)-4-(3,4,5-trimethoxyphenyl)butan-1-ol [20], the significant difference being the absence of signals for a methoxy group in the ^1^H and ^13^C NMR spectra. The flat ECD curve (Figure S38 in Supporting Information File 1) and nearly zero optical rotation of **3** (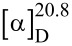 −1.34, *c* 0.28, MeOH) suggested that **3** was possibly a racemic mixture. Enantioseparation of **3** by HPLC using a chiral-pak IA column provided the enantiomers with a ratio about 3:2 (Figure S28, Supporting Information File 1) suggested its mixture feature. Unfortunately, the limited amount available of this compound did not allow the elucidation of its absolute configuration.

The remaining known compounds were identified as (+)-pinoresinol di-*O*-β-ᴅ-glucopyranoside (**4**) [21], dehydrodiconiferyl alcohol-4-*O*-β-ᴅ-glucopyranoside (**5**) [22], and lariciresinol-4-*O*-β-ᴅ-glucopyranoside (**6**) [23] (Figure 1) by comparing their physiochemical properties and spectral data with those reported in the literature.

### Biological activity

The isolated compounds were screened for their inhibitory effects on the LPS-induced NO production in RAW 264.7 macrophages. *N*^G^-Monomethyl-ʟ-arginine monoacetate salt (ʟ-NMMA, Sigma) was used as the positive control. As a result, compounds **1**, **2, 4**, and **5** showed mild inhibitory activities with inhibition rates in the range of 9.47–18.75%, compound **3** showed moderate activity with an inhibition rate of 42.06 ± 2.02% at a concentration of 50 μM (ʟ-NMMA, 59.31 ± 2.19%, Table 2).

**Table 2:** Inhibitory effects of compounds **1**–**6** on LPS-stimulated NO production.

sample	concentration (μM)	inhibition (%)

1	50	18.75 ± 2.13
2	50	69.16 ± 0.81 (cytotoxicity)
	12.5	15.93 ± 1.37
3	50	42.06 ± 2.02
4	50	9.47 ± 2.38
5	50	11.40 ± 0.81
6	50	3.36 ± 2.38
ʟ-NMMA^a^	50	59.31 ± 2.19

^a^Positive control.

## Conclusion

In summary, three new lignan derivatives, sinensiols H–J (**1**–**3**) and three known compounds (**4**–**6**), were obtained from the whole plants of *Selaginella sinensis*. The absolute configuration of compound **1** was established by comparison of calculated and experimental ECD spectra. Compounds **2** and **3**, which possess a 1,4-diphenylbutane skeleton, are rare examples of naturally occurring 9,9′-bisnorlignans. In in vitro bioassays, compound **3** was found to show a moderate inhibitory effect on NO production in LPS-induced RAW 264.7 cells with an inhibitory rate of 42.06 ± 2.02% at 50 μM.

## Experimental

### General experimental procedures

Optical rotations were carried out on an Autopol VI automatoc polarimeter. UV spectra were recorded on a Shimadzu UV-2401 PC spectrophotometer. IR spectra (KBr) were determined on a Bruker Vertex 70 infrared spectrometer. ESI and HRESIMS were performed on an UPLCIT-TOF spectrometer. ECD spectra were obtained on a Chirascan-plus CD spectrometer (Applied Photophysics Ltd., UK). NMR spectral data were measured on a Bruker DRX-600 spectrometer. Silica gel (200–300 mesh, Qingdao Haiyang Chemical Co. Ltd., China) was used for column chromatography. Semi-preparative HPLC was performed on an Agilent 1260 liquid chromatograph with a Zorbax SB-C18 (9.4 mm × 150 mm) column.

### Plant material

*Selaginella sinensis* was collected from Luoyang, Henan, China, in April 2021 and identified by Prof. Liang Zhang (Kunming Institute of Botany, CAS). A voucher specimen (No. 20210412) has been deposited in the school of pharmacy, Guizhou Medical University.

### Extraction and isolation

The air-dried powder of the whole plants of *S. sinensis* (5.2 kg) was extracted three times with 95% ethanol at room temperature. The combined extracts were concentrated and yielded 423 g of a crude extract which was subjected to reversed-phase MPLC (MCI; MeOH/H_2_O, 5–95%, v/v) to give fractions 1–5. Fr. 2 (58 g) was subjected to silica gel column chromatography (CC) eluting with CH_2_Cl_2_/MeOH 9:1 to yield six major fractions (1–6). Fr. 2.2 (0.5 g) then was further purified by preparative HPLC (MeOH/H_2_O 28:72) to afford compound **5** (20.5 mg) and compound **6** (13.7 mg). Fr. 2.5 (7.50 g) was further purified by silica gel CC with (CH_2_Cl_2_/MeOH 9:1) to give compound **4** (120.5 mg). Fr. 3 (37 g) was further purified by reversed-phase chromatography (RP-18 column) using MeOH/H_2_O 4:6 to afford compound **1** (4.8 mg). Fr. 4 (21 g) was chromatographed on a silicagel column eluting with a CH_2_Cl_2_/MeOH gradient system (v/v = 30:1–9:1) to give 8 fractions (Fr. 4.1–Fr. 4.8). Fr. 4.3 was separated by silica gel column chromatography and purified by semipreparative HPLC (3 mL/min) using MeOH/H_2_O 45:55 to give compounds **2** (5.3 mg) and **3** (2.6 mg).

### Identification of new compounds

**Compound 1**: pale yellow amorphous powder, 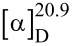 27.81 (*c* 0.32, MeOH); IR (KBr) ν_max_: 3540, 1764, 1515, 1445, 1247, 1035 cm^−1^; ECD (*c* 2.2 × 10^−4^ M, MeOH) λ_max_ (Δε) 204 (+4.36), 231 (+1.79) nm; ^1^H and ^13^C NMR data, see Table 1; HRESIMS (*m*/*z*): [M − H]^−^ calcd for C_20_H_19_O_7_, 371.1136; found, 371.1133.

**Compound 2**: white amorphous powder, 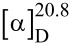 −1.35 (*c* 0.24, MeOH); IR (KBr) ν_max_: 3417, 1593, 1510, 1350, 1104 cm^−1^; ^1^H and ^13^C NMR data, see Table 1; HRESIMS (*m*/*z*): [M − H]^−^ calcd for C_20_H_23_O_6_, 359.1500; found, 359.1497.

**Compound 3**: white amorphous powder, 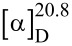 −1.34 (*c* 0.28, MeOH); IR (KBr) ν_max_: 3433, 2937.2, 1593.0, 1241.6, 814.9 cm^−1^; ^1^H and ^13^C NMR data, see Table 1; HRESIMS (*m*/*z*): [M + COOH]^−^ calcd for C_20_H_23_O_8_, 391.1398; found, 391.1394.

### Nitric oxide production inhibitory assay

The inhibitory activity against the production of NO was evaluated using LPS induced RAW 264.7 cells as previously reported [24]. The cells were seeded in 96-well plates and co-incubated with the test compounds and positive control drug at a concentration of 50 μM or 12.5 μM, followed by stimulation with 1 μg/mL LPS for 18 h. The viability of RAW 264.7 cells was determined by an MTS assay to exclude the interference of the cytotoxicity of the test compounds before the nitric oxide (NO) production assay. NO production in each well was assessed by measuring the accumulation of nitrite in the culture supernatants using Griess reagent. After 5 min of incubation, the absorbance was measured using a microplate reader (Thermo, Bio-rad, USA) at 570 nm. ʟ-NMMA was used as the positive control. Experiments were operated in triplicate. All values are described as mean ± SD of three independent experiments.

## Supporting Information

File 1 ECD calculation method of compound **1** and HPLC analysis of **3** and NMR, MS, and IR spectra of compounds **1**–**3**.
