# Supplementary material for: Sinensiols H–J, three new lignan derivatives from Selaginella sinensis (Desv.) Spring
Source: Beilstein J Org Chem. 2022 Oct 7;18:1410–5. doi: 10.3762/bjoc.18.146 (PMC9551277; doi:10.3762/bjoc.18.146)
Supplement: File 1 — ECD calculation method of compound 1 and HPLC analysis of 3 and NMR, MS, and IR spectra of compounds 1–3. [file Beilstein_J_Org_Chem-18-1410-s001.pdf]

BEILSTEIN JOURNAL OF ORGANIC CHEMISTRY

Supporting Information

for

Sinensiols H–J, three new lignan derivatives from *Selaginella sinensis* (Desv.) Spring

Qinfeng Zhu, Beibei Gao, Qian Chen, Tiantian Luo, Guobo Xu and Shanggao Liao

Beilstein J. Org. Chem. **2022,** *18,* 1410–1415. doi:10.3762/bjoc.18.146

ECD calculation method of compound 1 and HPLC analysis of 3 and NMR, MS, and IR spectra of compounds 1–3

License and Terms: This is a supporting information file under the terms of the Creative Commons Attribution License (https://creativecommons.org/licenses/by/4.0). Please note that the reuse, redistribution and reproduction in particular requires that the author(s) and source are credited and that individual graphics may be subject to special legal provisions.
The license is subject to the *Beilstein Journal of Organic Chemistry* terms and conditions: (https://www.beilstein-journals.org/bjoc/terms)

Table of contents	Page
**Figure S1**. ^{1}H NMR spectrum of **1** in DMSO-d_6	S2
**Figure S2**. ^{13}C NMR spectrum of **1** in DMSO-d_6	S2
**Figure S3**. HSQC spectrum of **1** in DMSO-d_6	S3
**Figure S4**. HMBC spectrum of **1** in DMSO-d_6	S3
**Figure S5**. ^{1}H-^{1}H COSY spectrum of **1** in DMSO-d_6	S4
**Figure S6**. ROESY spectrum of **1** in DMSO-d_6	S4
**Figure S7**. ^{1}H NMR spectrum of **1** in $CDCl_3$	S5
**Figure S8**. ^{13}C NMR spectrum of **1** in $CDCl_3$	S5
**Figure S9**. HSQC spectrum of **1** in $CDCl_3$	S6
**Figure S10**. HMBC spectrum of **1** in $CDCl_3$	S6
**Figure S11.** ^{1}H-^{1}H COSY spectrum of **1** in $CDCl_3$	S7
**Figure S12.** ROESY spectrum of **1** in $CDCl_3$	S7
**Figure S13.** HRESIMS spectrum of **1**	S8
**Figure S14.** IR spectrum of **1**	S9
**Figure S15.** UV spectrum of **1**	S9
**Figure S16.** Experimental ECD curves of compounds **1**	S10
**ECD calculation method of compound 1**	
**Figure S17.** Experimental and calculated ECD spectra of **1**	S10
**Figure S18**. Optimized lowest energy conformers for (8*R*,8'*S*)-**1** at B3LYP/6-31G(d) level.	S11
**Table S1**. Energy (298.15 K) analysis for (8*R*,8'*S*)-**1**.	S11
**Table S2.** Calculated ECD data for (8*R*,8'*S*)-**1**	S12
**Figure S19.** ^{1}H NMR spectrum of **2** in MeOH-d_4	S12
**Figure S20.** ^{13}C NMR spectrum of **2** in MeOH-d_4	S13
**Figure S21.** HSQC spectrum of **2** in MeOH-d_4	S13
**Figure S22.** HMBC spectrum of **2** in MeOH-d_4	S14
**Figure S23.** ^{1}H-^{1}H COSY spectrum of **2** in MeOH-d_4	S14
**Figure S24.** ROESY spectrum of **2** in MeOH-d_4	S15
**Figure S25.** HRESIMS spectrum of **2**	S16
**Figure S26.** IR spectrum of **2**	S17
**Figure S27.** UV spectrum of **2**	S17
**Figure S28.** HPLC analysis of **3** by a chiral-pak IA column	S18
**Figure S29.** ^{1}H NMR spectrum of **3** in MeOH-d_4	S19
**Figure S30.** ^{13}C NMR spectrum of **3** in MeOH-d_4	S19
**Figure S31.** HSQC spectrum of **3** in MeOH-d_4	S20
**Figure S32.** HMBC spectrum of **3** in MeOH-d_4	S20
**Figure S33.** ^{1}H-^{1}H COSY spectrum of **3** in MeOH-d_4	S21
**Figure S34.** ROESY spectrum of **3** in MeOH-d_4	S21
**Figure S35.** HRESIMS spectrum of **3**	S22
**Figure S36.** IR spectrum of **3**	S23
**Figure S37.** UV spectrum of **3**	S23
**Figure S38.** Experimental ECD curves of compounds **3**	S24

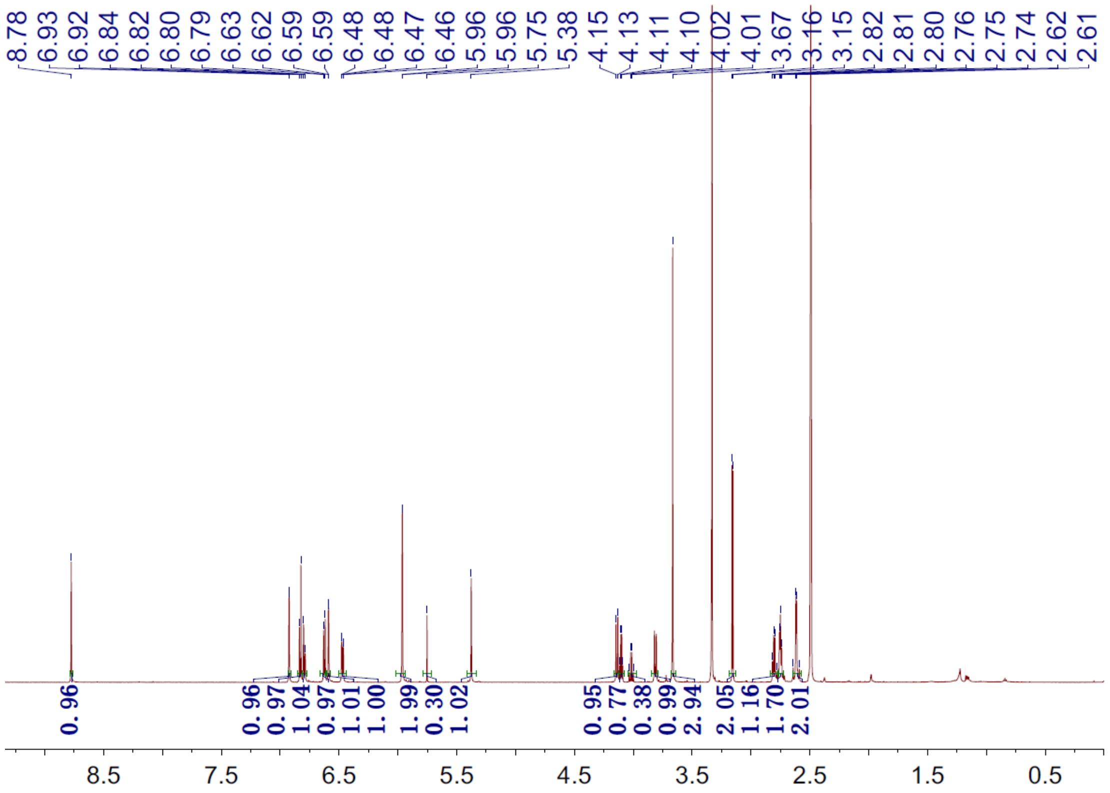

Figure S1. ^{1}H NMR spectrum of **1** in DMSO-d_6

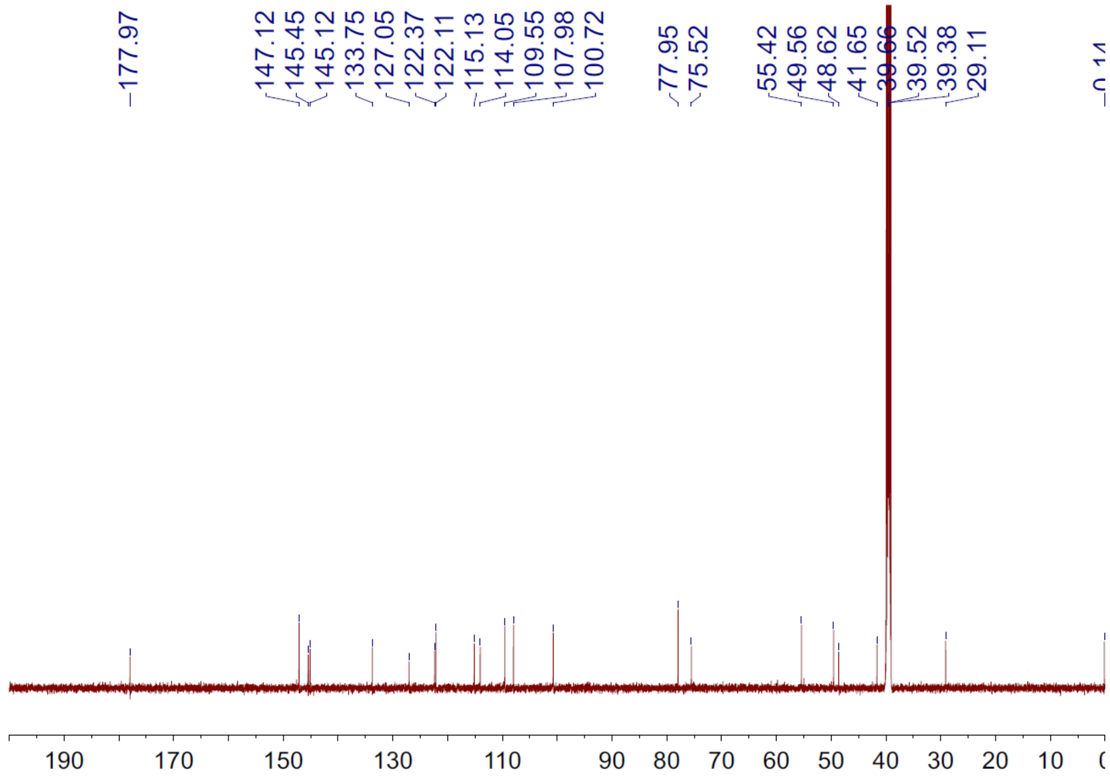

Figure S2. ^{13}C NMR spectrum of **1** in DMSO-d_6

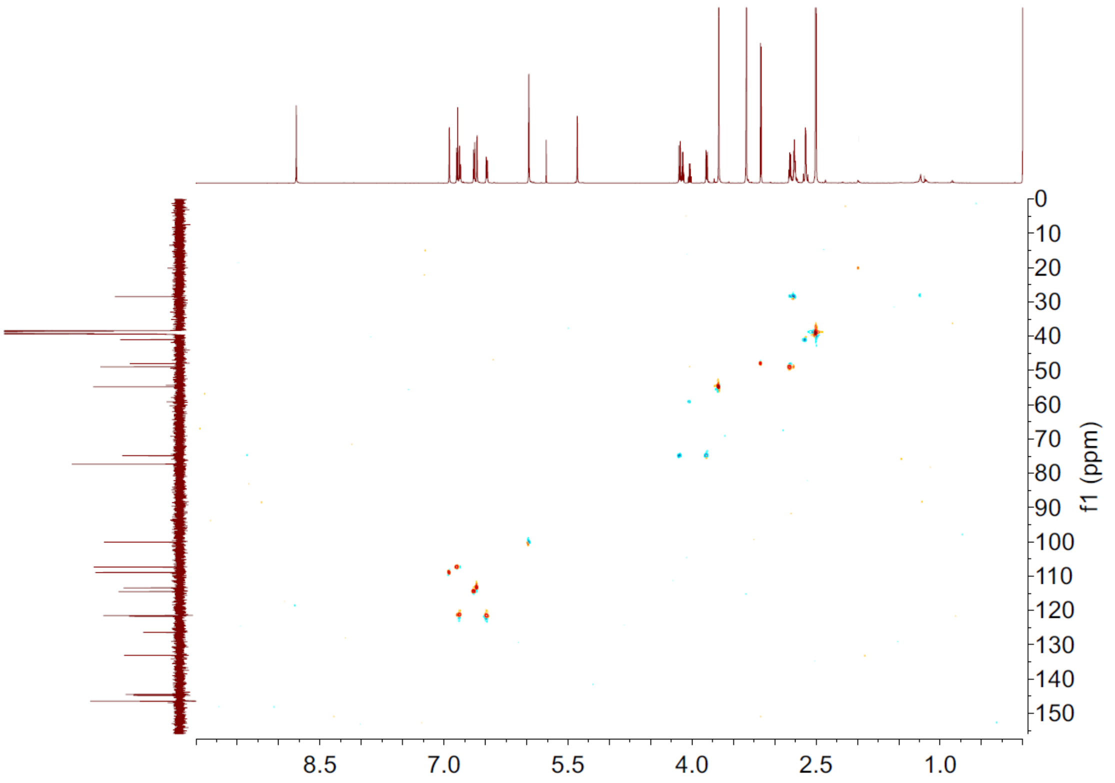

Figure S3. HSQC spectrum of **1** in DMSO-d_6

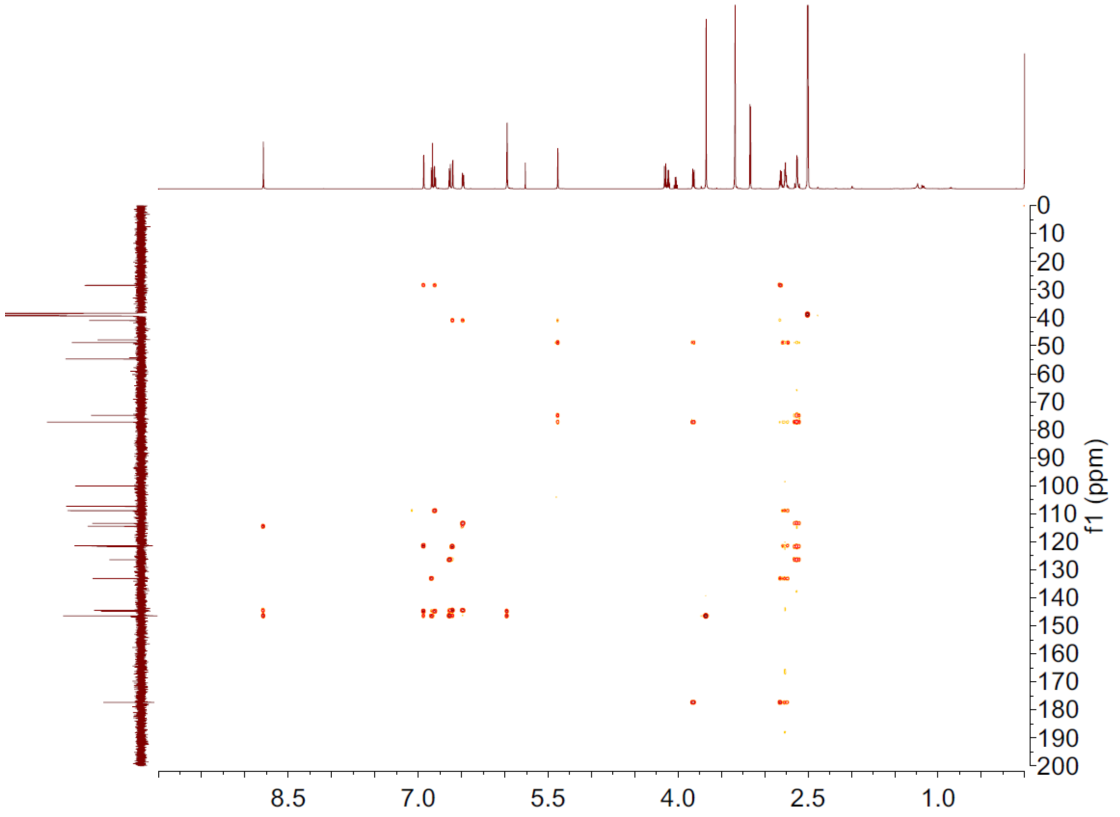

Figure S4. HMBC spectrum of **1** in DMSO-d_6

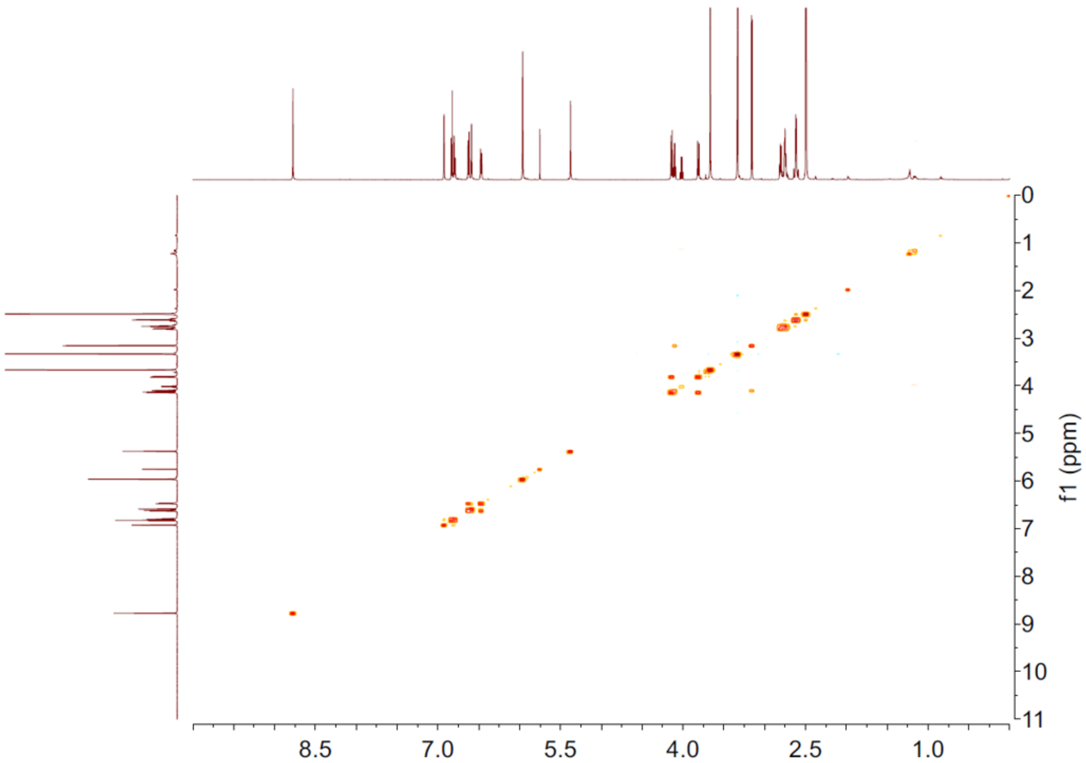

Figure S5. ^{1}H-^{1}H COSY spectrum of **1** in DMSO-d_6

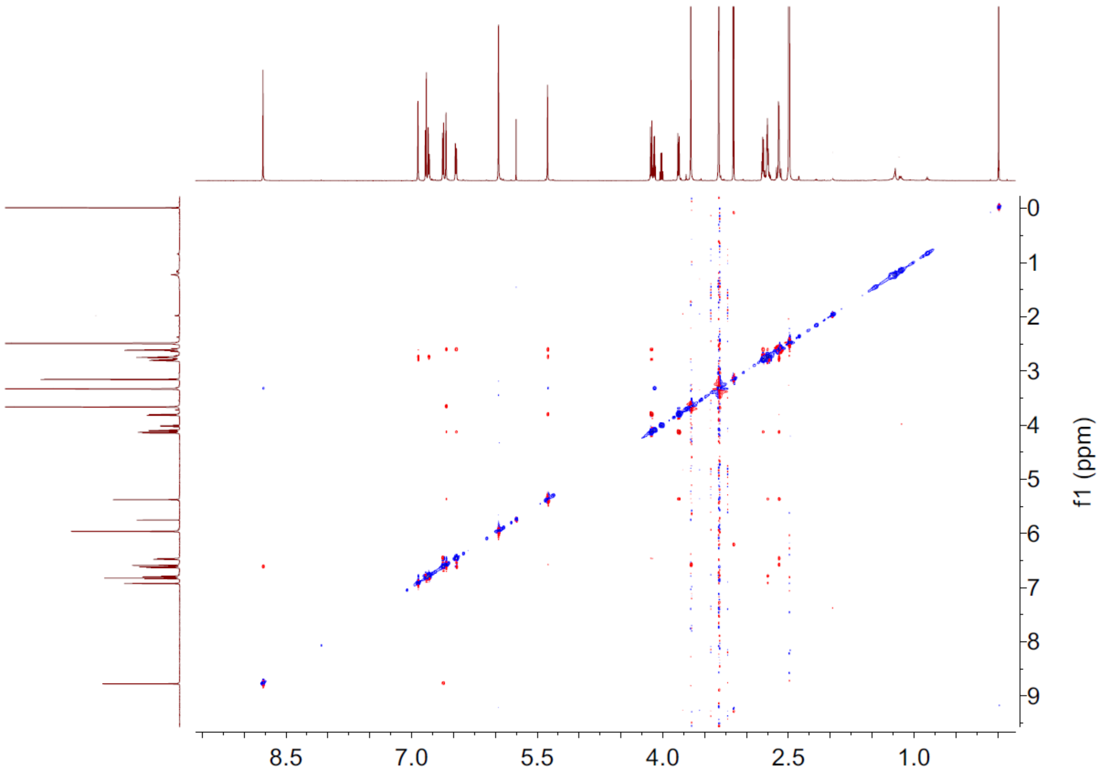

Figure S6. ROESY spectrum of **1** in DMSO-d_6

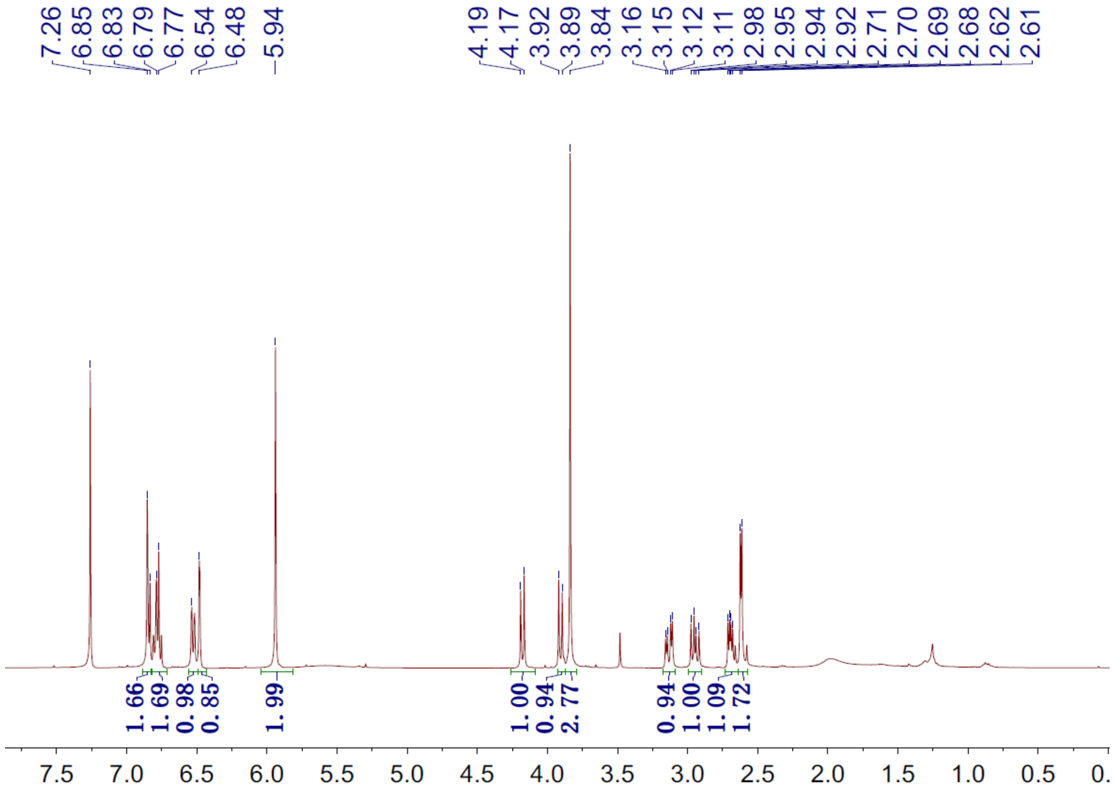

Figure S7. ^{1}H NMR spectrum of **1** in $CDCl_3$

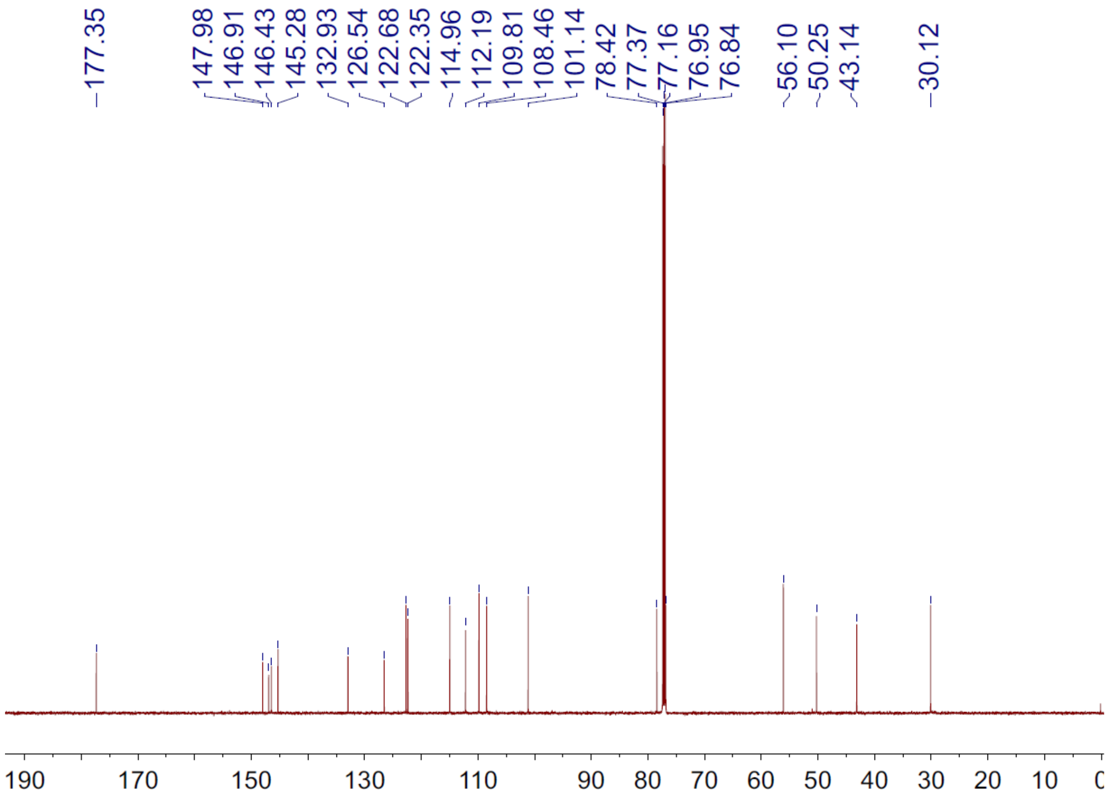

Figure S8. ^{13}C NMR spectrum of **1** in $CDCl_3$

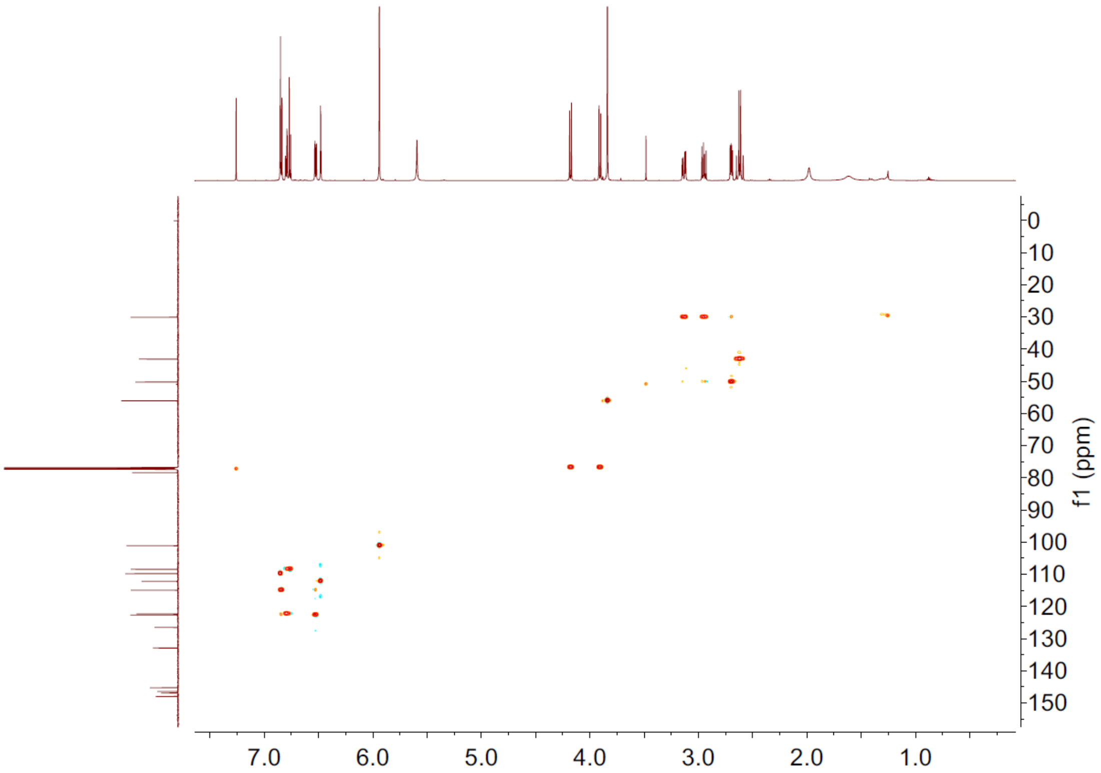

Figure S9. HSQC spectrum of **1** in $CDCl_3$

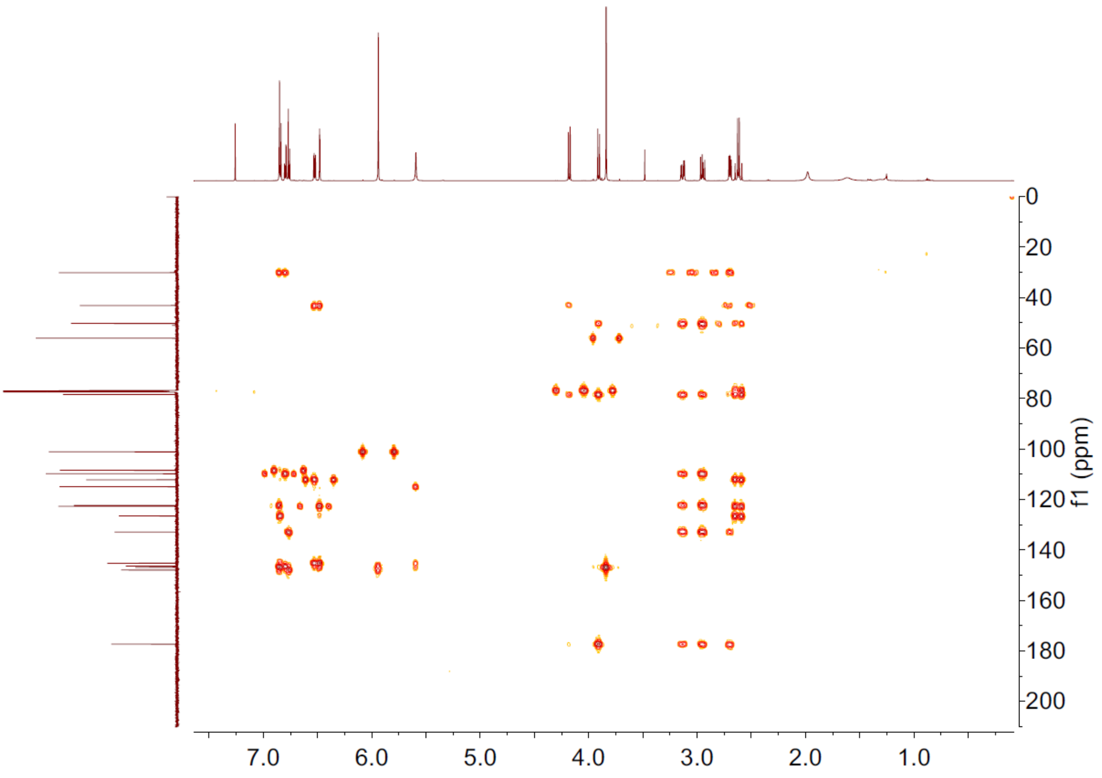

Figure S10. HMBC spectrum of **1** in $CDCl_3$

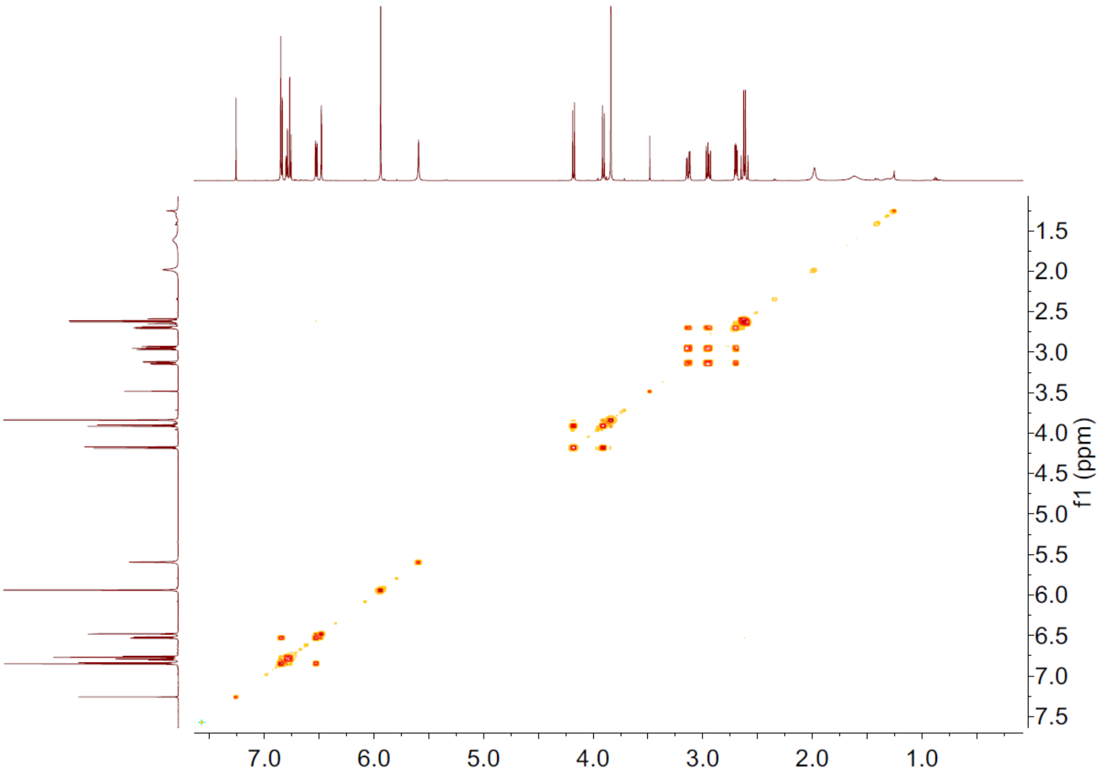

Figure S11. ^{1}H-^{1}H COSY spectrum of **1** in $CDCl_3$

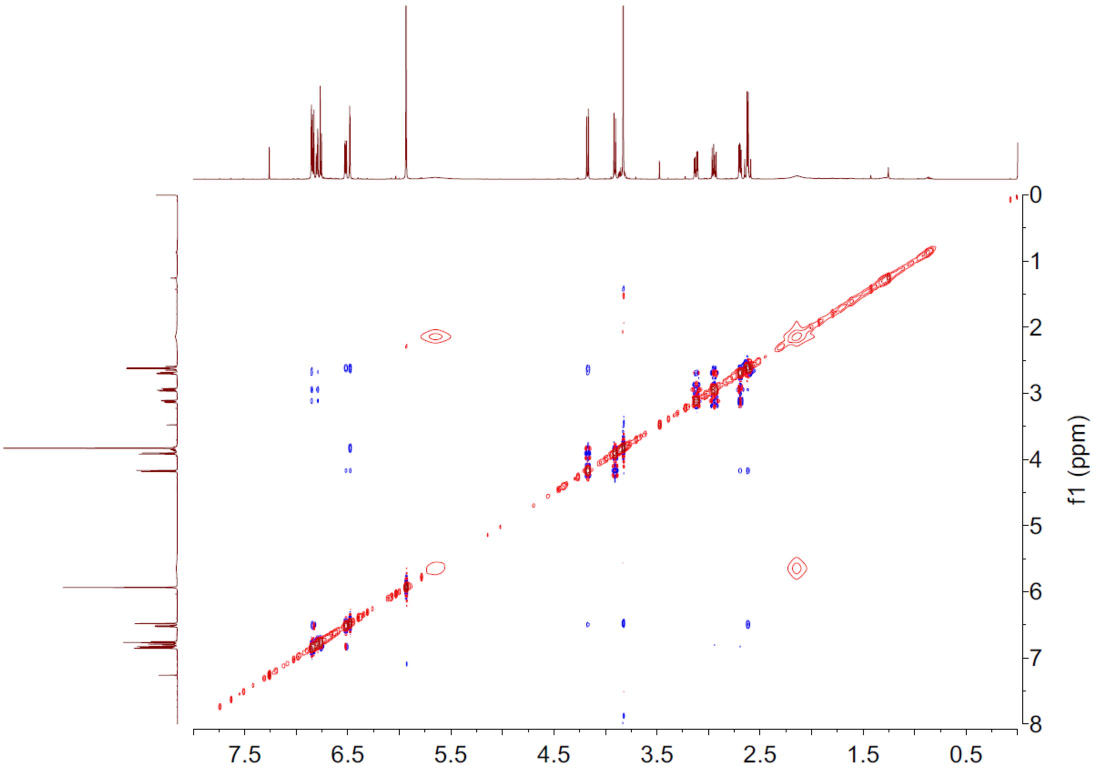

Figure S12. ROESY spectrum of **1** in $CDCl_3$

Qualitative Analysis Report

**Data Filename**	fzh-27.d	**Sample Name**	fzh-27
**Sample Type**	Sample	**Position**	P1-A4
**Instrument Name**	Instrument 1	**User Name**	
**Acq Method**	s-.m	**Acquired Time**	4/15/2022 4:25:37 PM
**IRM Calibration Status**	Success	**DA Method**	PCDL.m
**Comment**			

Sample Group **Info.**
Acquisition SW 6200 series TOF/6500 series
Version Q-TOF B.05.01 (B5125.2)

User Spectra

Fragmentor Voltage	Collision Energy	Ionization Mode
135	0	ESI

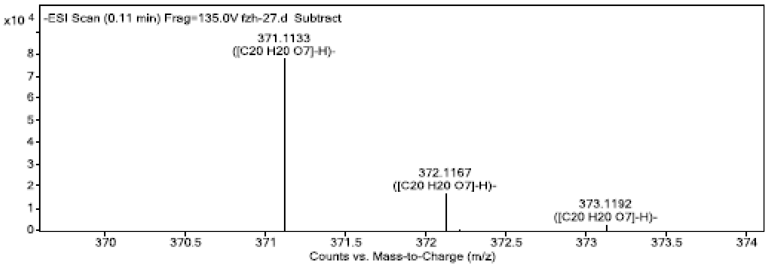

Peak List

m/z	z	Abund	Formula	Ion
112.9856	1	24469.66		
341.1083	1	3080.53		
371.1133	1	77806.63	C20 H20 O7	(M-H)-
372.1167	1	16677.76	C20 H20 O7	(M-H)-
407.0884	1	4511.99		
417.1186	1	21906.09		
418.1235	1	5608.64		
485.1062	1	12670.79		
1033.9881	1	14093.31		
1034.9904	1	6341.52		

Formula Calculator Element Limits

Element	Min	Max
C	3	60
H	0	120
O	0	30

Formula Calculator Results

Formula	CalculatedMass	CalculatedMz	Mz	Diff. (mDa)	Diff. (ppm)	DBE
C20 H20 O7	372.1209	371.1136	371.1133	0.30	0.81	11.0000

--- End Of Report ---

Figure S13. HRESIMS spectrum of **1**

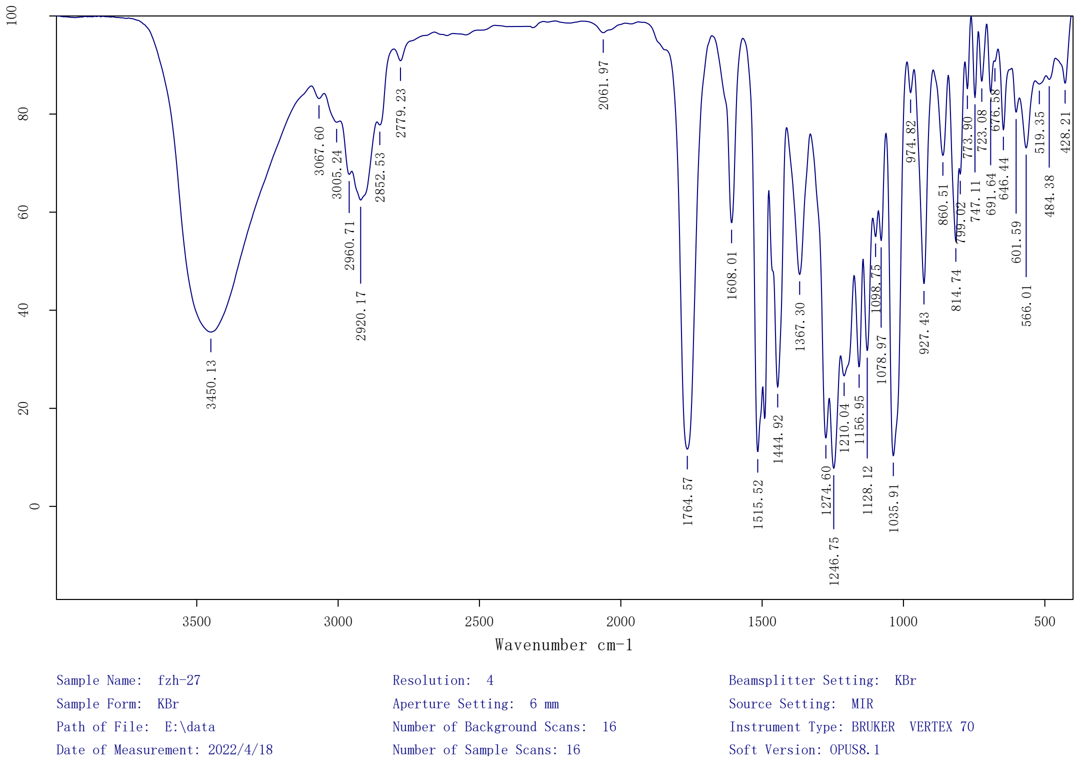

Figure S14. IR spectrum of **1**

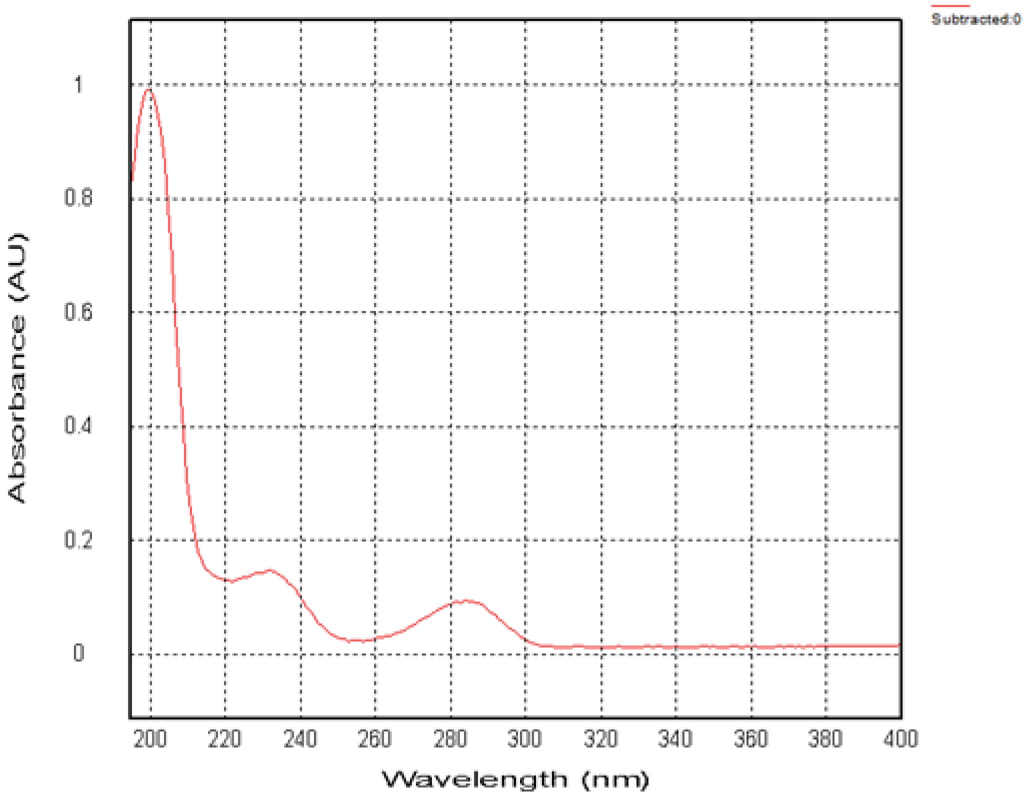

Figure S15. UV spectrum of **1**

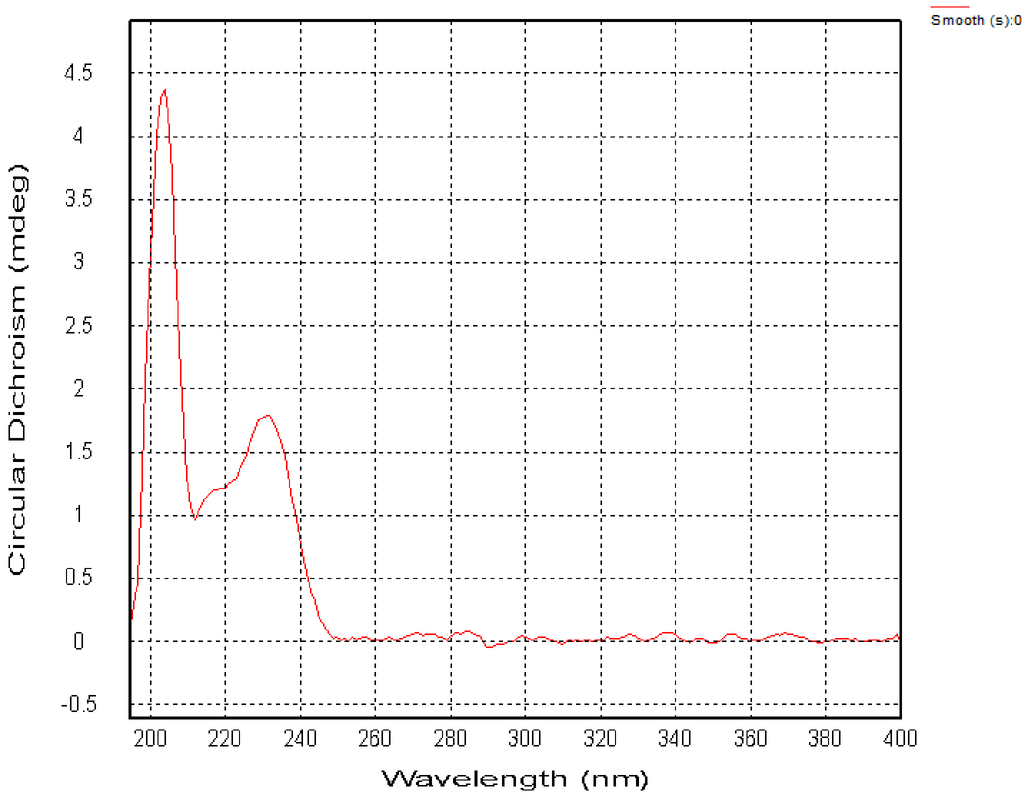

Figure S16. Experimental ECD curves of compounds **1**

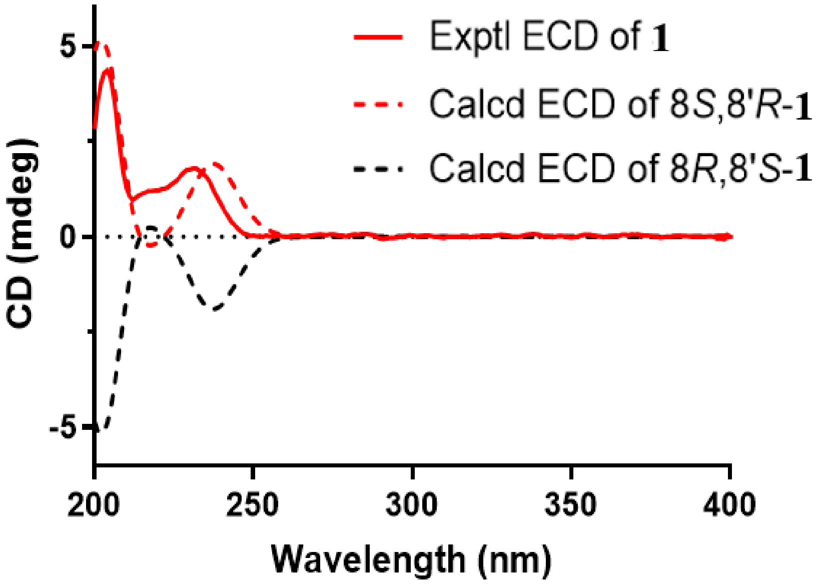

Figure S17. Experimental and calculated ECD spectra of **1**

The absolute configuration of **1** was studied by comparison of its experimental and simulated electronic circular dichroism (ECD) spectra using the quantum chemical time-dependent density functional theory (TDDFT)

method. As shown in Figure S17, the experimental ECD spectrum of **1** showed two significant positive Cotton effects around 204 and 231 nm, which matched well with those calculated for the (8*S*,8'*R*)-**1**, indicating that **1** possessed the same corresponding absolute configuration. Details of ECD calculations were provided as following:

ECD calculation method of compound 1

Conformational analyses of (8*R*,8'*S*)-**1** were first carried out in the SYBYL-X-2.1.1 program using MMFF94s molecular force field. The stable conformers were optimized at b3lyp/6-31G(d) in the gas phase and further subjected to ECD calculations at cam-b3lyp/6-31+g(d,p) level in the PCM model of methanol using Gaussian09 program. The simulated spectra of the lowest energy conformers were averaged according to the Boltzmann distributions, and generated using SpedDis software. The spectra of (8*S*,8'*R*)-**1** were generated using the enantiomeric ECD operations by SpedDis.

Conformers and energy analysis

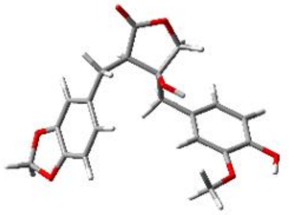

1-1

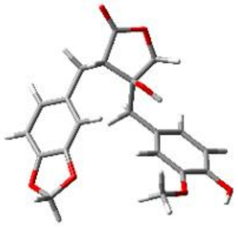

1-2

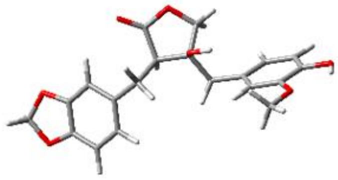

1-3

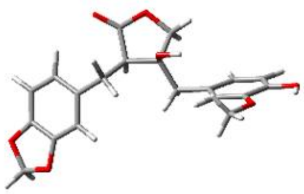

1-4

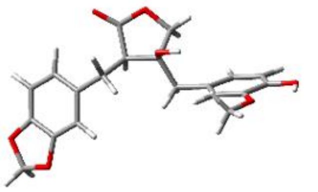

1-5

Figure S18. Optimized lowest energy conformers for (8*R*,8'*S*)-**1** at B3LYP/6-31G(d) level.

Table S1. Energy (298.15 K) analysis for (8*R*,8'*S*)-**1**.

conformer	Energy (Hartree)	Energy (kcal/mol)	Population (%)
1-1	-1300.215311	-815897.4193	49.31
1-2	-1300.214398	-815896.8462	18.75
1-3	-1300.214228	-815896.74	15.67
1-4	-1300.21361	-815896.3518	8.14
1-5	-1300.213609	-815896.3514	8.13

Table S2. Calculated ECD data for (8*R*,8'*S*)-**1**

	1-1		1-2		1-3		1-4		1-5	
states	excitation energies	rotatory strengths	excitation energies	rotatory strengths	excitation energies	rotatory strengths	excitation energies	rotatory strengths	excitation energies	rotatory strengths
1	4. 8597	-25. 0782	4. 9019	-11. 2136	4. 8841	-5. 291	4. 8941	-18. 1508	4. 8941	-18. 1516
2	5. 0012	2. 3065	5. 0156	-1. 3713	5. 0011	-4. 5018	5. 0017	7. 0747	5. 0017	7. 0918
3	5. 5213	-14. 2497	5. 4631	19. 7397	5. 5386	3. 6457	5. 4917	-1. 6252	5. 4917	-1. 6386
4	5. 5327	1. 4241	5. 5304	-0. 1686	5. 5602	-5. 818	5. 5353	19. 1049	5. 5353	19. 1228
5	5. 5685	7. 6587	5. 6062	-16. 2483	5. 5785	10. 0594	5. 5391	1. 7255	5. 5391	1. 7175
6	5. 6277	-34. 7902	5. 6237	-28. 5398	5. 6253	-46. 4445	5. 6235	-42. 6728	5. 6235	-42. 6809
7	5. 9001	-2. 2655	5. 8911	-13. 6475	5. 9041	-18. 0581	5. 8987	-17. 6945	5. 8986	-17. 6821
8	6. 1	-18. 256	6. 1322	-25. 0213	6. 0991	-14. 2771	6. 0993	-13. 5084	6. 0992	-13. 5068
9	6. 2185	5. 0354	6. 199	-0. 1103	6. 1471	1. 1297	6. 145	-0. 7125	6. 145	-0. 7134
10	6. 2282	6. 256	6. 2786	3. 2449	6. 2735	-3. 7759	6. 2794	-1. 0819	6. 2794	-1. 0822

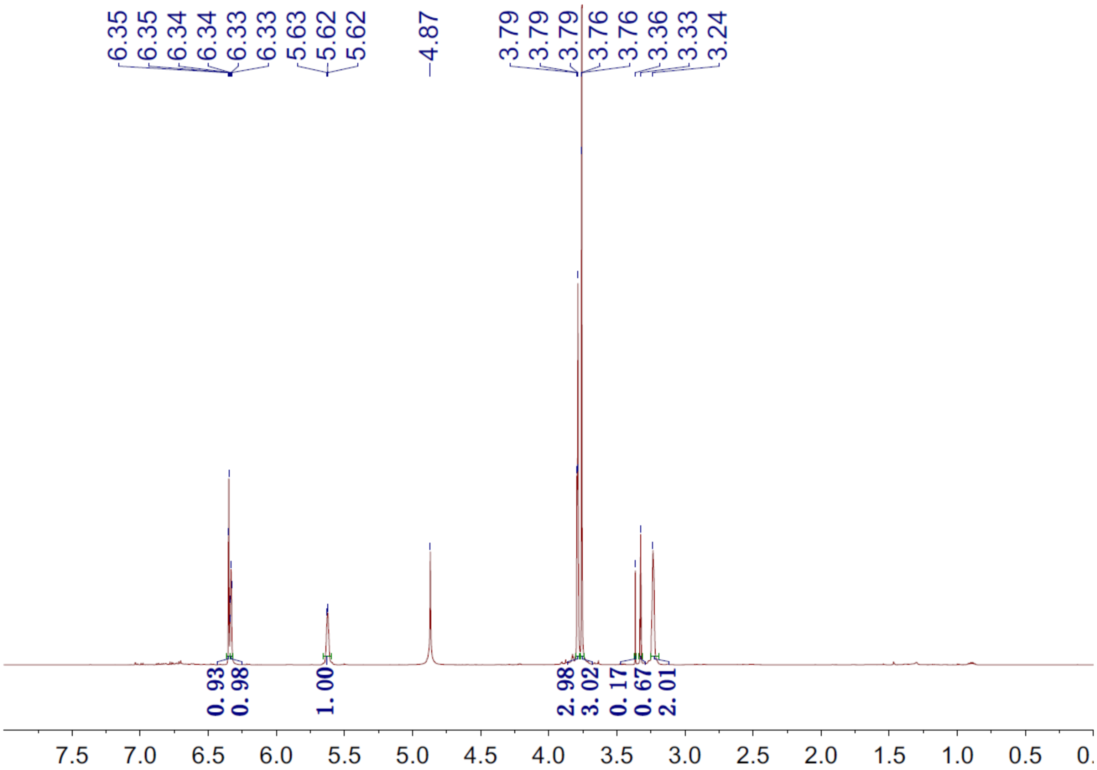

Figure S19. ^{1}H NMR spectrum of **2** in MeOH-d_4

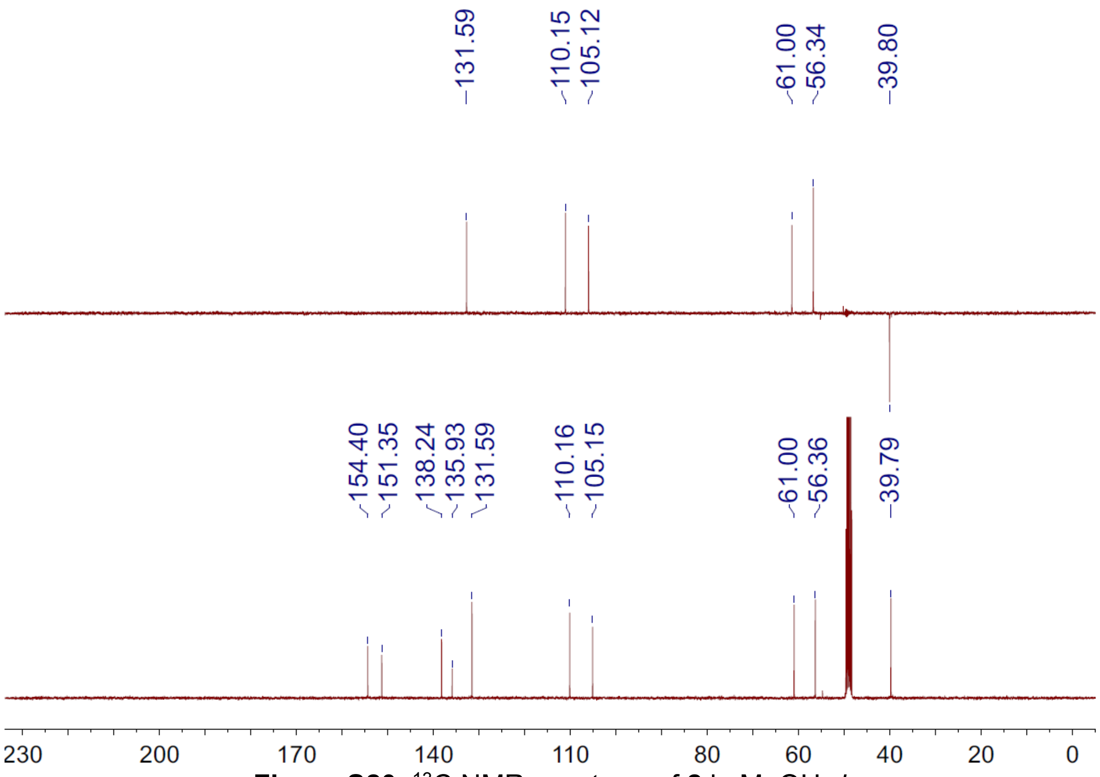

Figure S20. ^{13}C NMR spectrum of **2** in MeOH-d_4

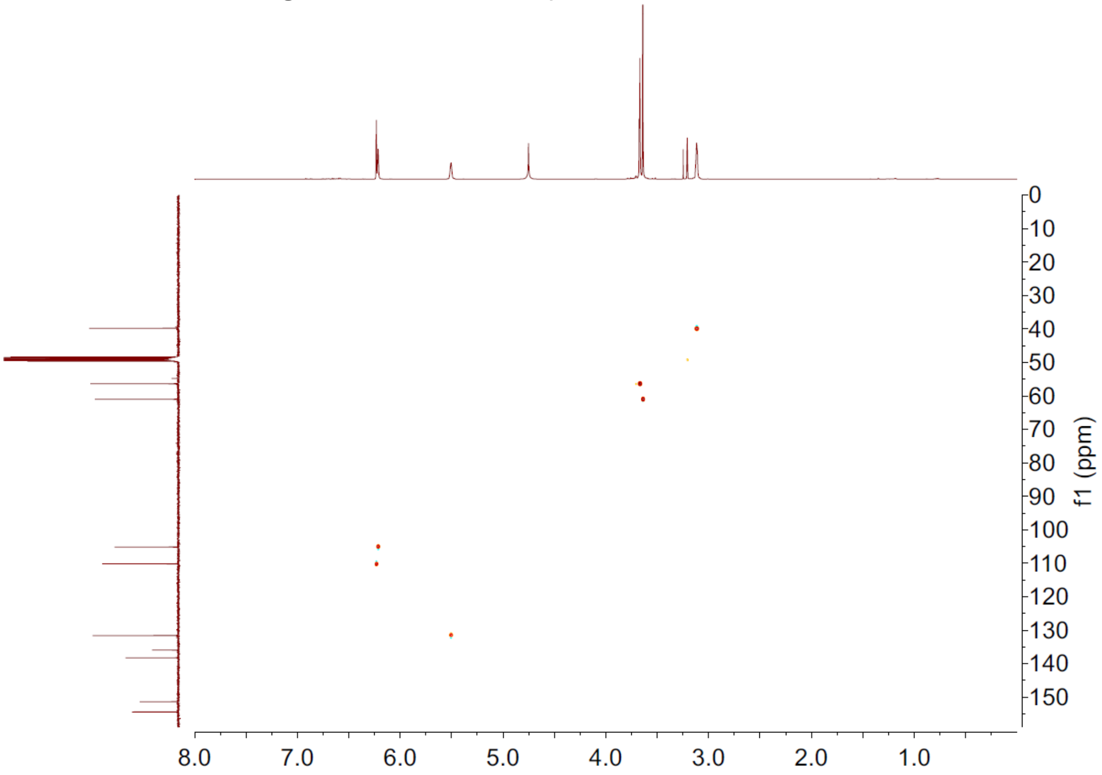

Figure S21. HSQC spectrum of **2** in MeOH-d_4

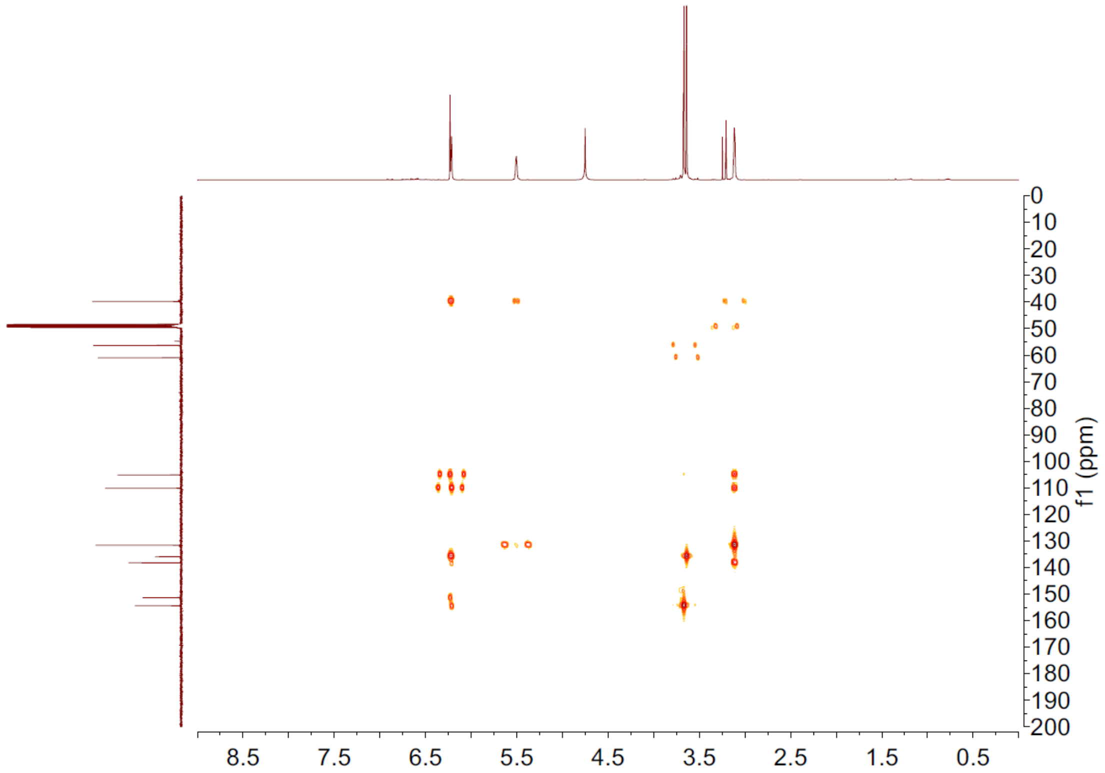

Figure S22. HMBC spectrum of **2** in MeOH-d_4

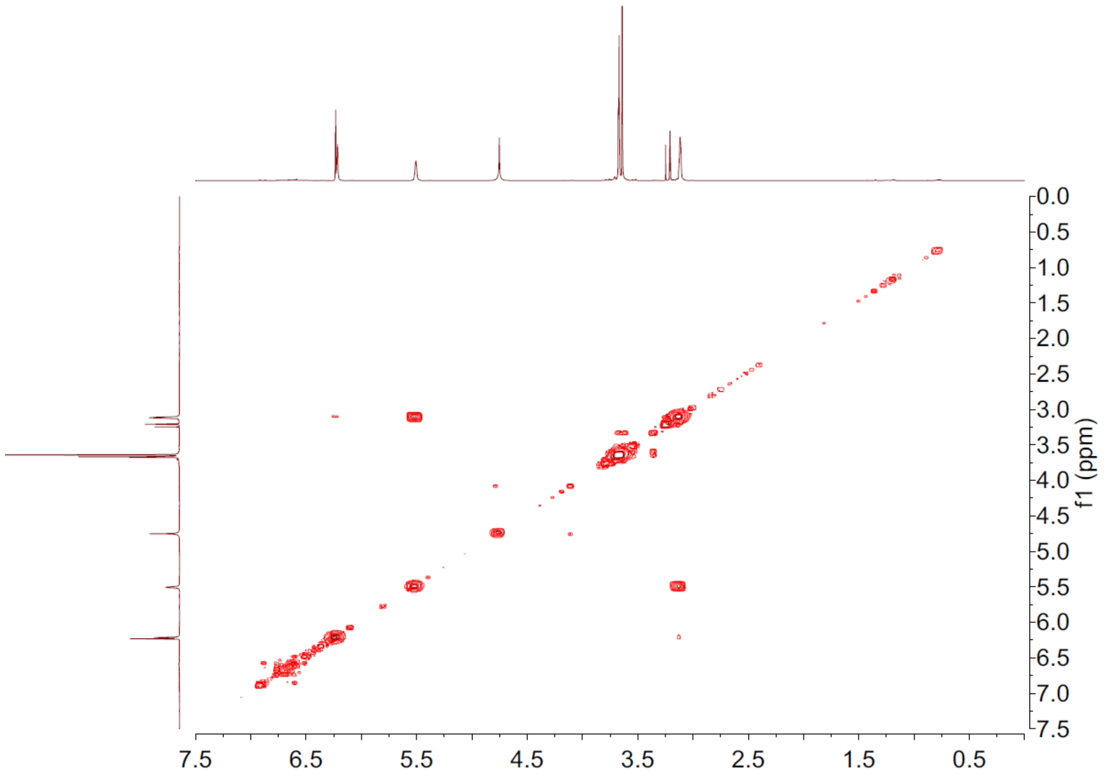

Figure S23. ^{1}H-^{1}H COSY spectrum of **2** in MeOH-d_4

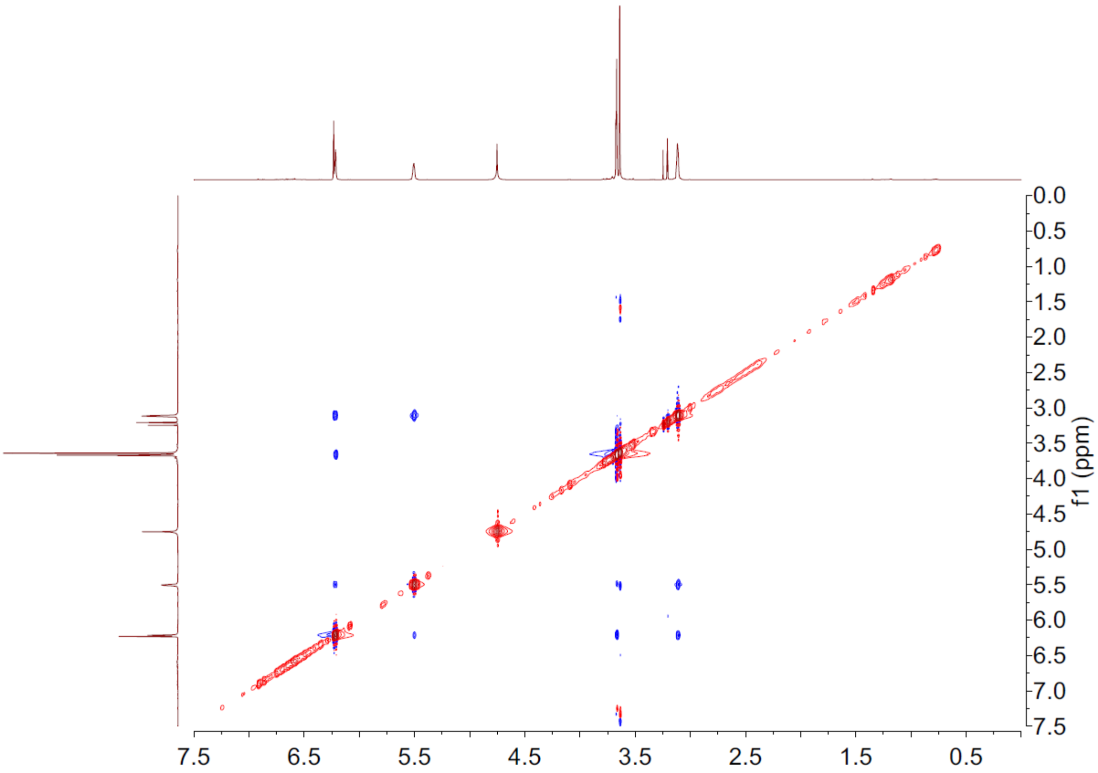

Figure S24. ROESY spectrum of **2** in MeOH-d_4

Qualitative Analysis Report

**Data Filename**	fzh-25.d	**Sample Name**	fzh-25
**Sample Type**	Sample	**Position**	P1-A3
**Instrument Name**	Instrument 1	**User Name**	
**Acq Method**	s-.m	**Acquired Time**	4/15/2022 4:24:27 PM
**IRM Calibration Status**	Success	**DA Method**	PCDL.m
**Comment**			

**Sample Group**		**Info.**
**Acquisition SW Version**	6200 series TOF/6500 series Q-TOF B.05.01 (B5125.2)	

User Spectra

Fragmentor Voltage	Collision Energy	Ionization Mode
135	0	ESI

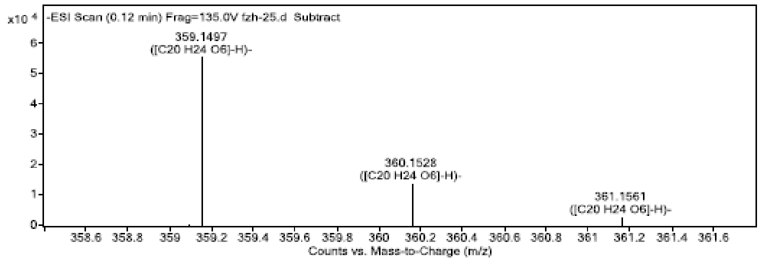

Peak List

m/z	z	Abund	Formula	Ion
68.9958	1	4582.38		
89.0244	1	3589.3		
112.9856	1	4662.08		
248.96	1	7709.95		
359.1497	1	55558.7	C20 H24 O6	(M-H)-
360.1528	1	13890.06	C20 H24 O6	(M-H)-
391.1395	1	5741.64		
495.2028	1	3120.57		
681.2955	1	4889.1		
1034.9905	1	3900.38		

Formula Calculator Element Limits

Element	Min	Max
C	3	60
H	0	120
O	0	30

Formula Calculator Results

Formula	CalculatedMass	CalculatedMz	Mz	Diff. (mDa)	Diff. (ppm)	DBE
C20 H24 O6	360.1573	359.1500	359.1497	0.30	0.84	9.0000

--- End Of Report ---

Figure S25. HRESIMS spectrum of **2**

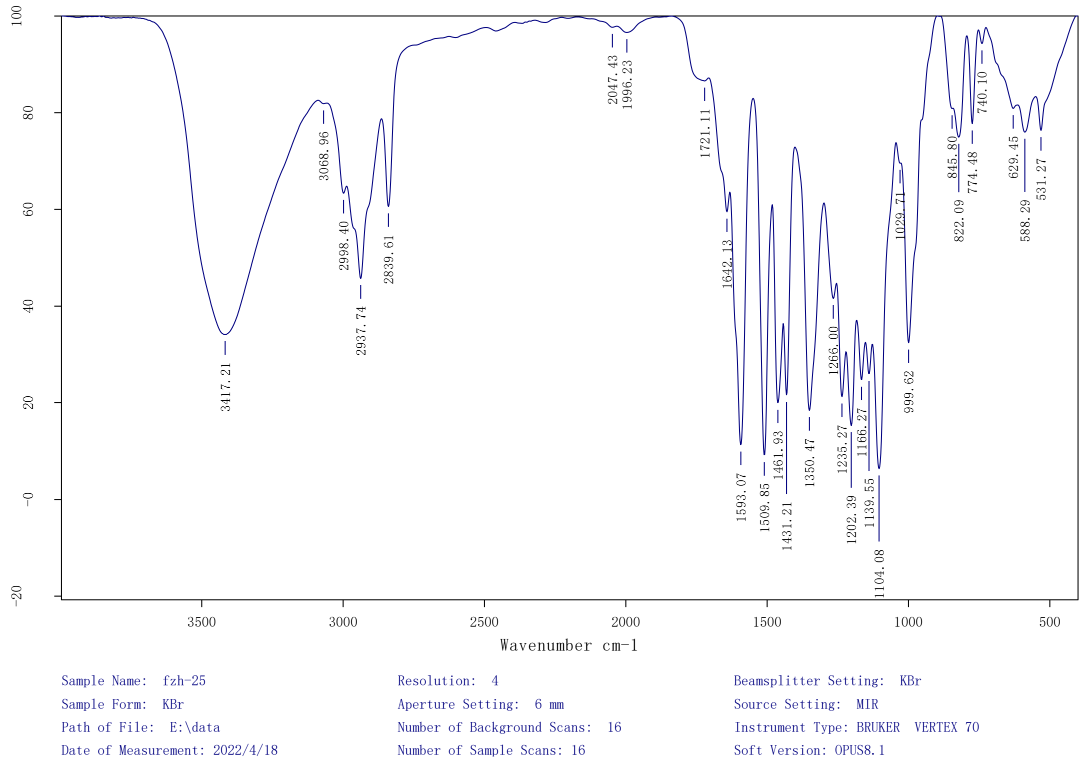

Figure S26. IR spectrum of **2**

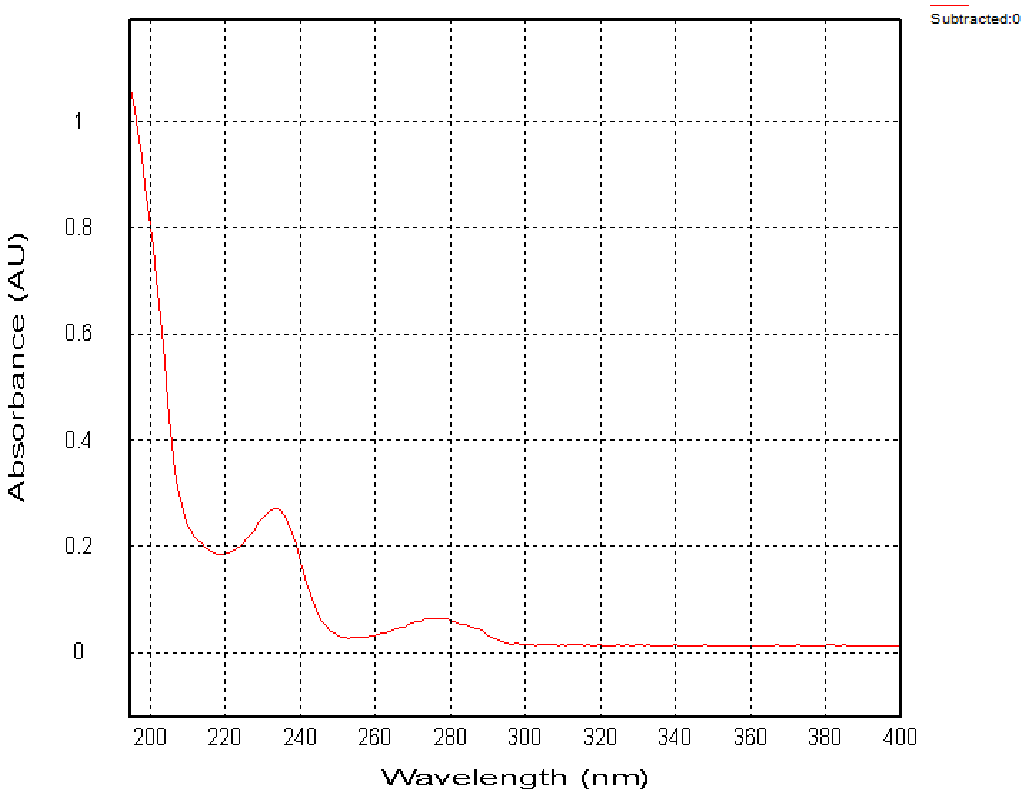

Figure S27. UV spectrum of **2**

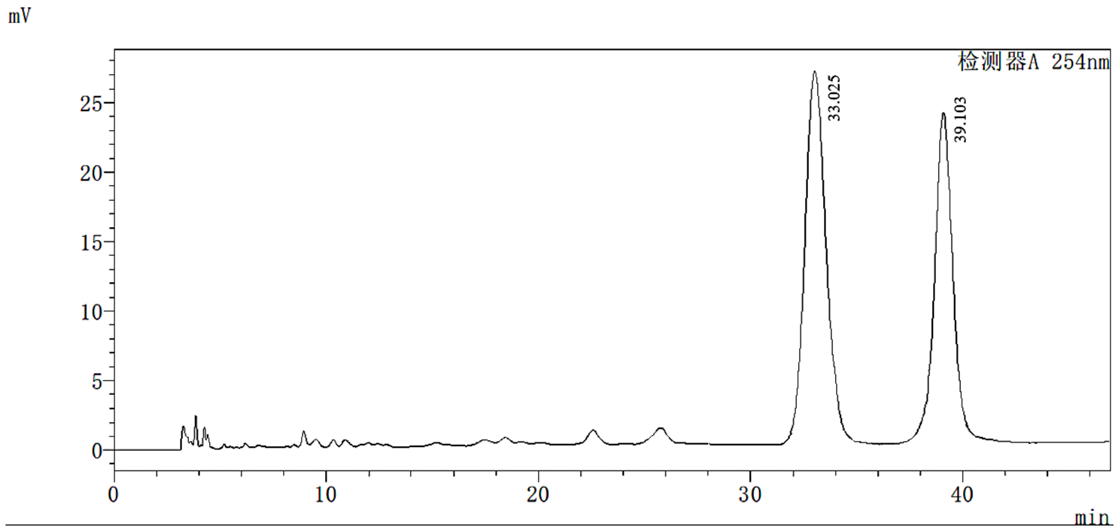

No.	retention time	peak height	peak area	Result (%)	computed result (%)
1	33.025	24310	1479329	60.986	60.986
2	39.103	19795	946285	39.012	39.012

Figure S28. HPLC analysis of **3** by a chiral-pak IA column (mobile phase: *n*-hexane/isopropyl alcohol 85:1, v/v, flow rate: 1 mL/min).

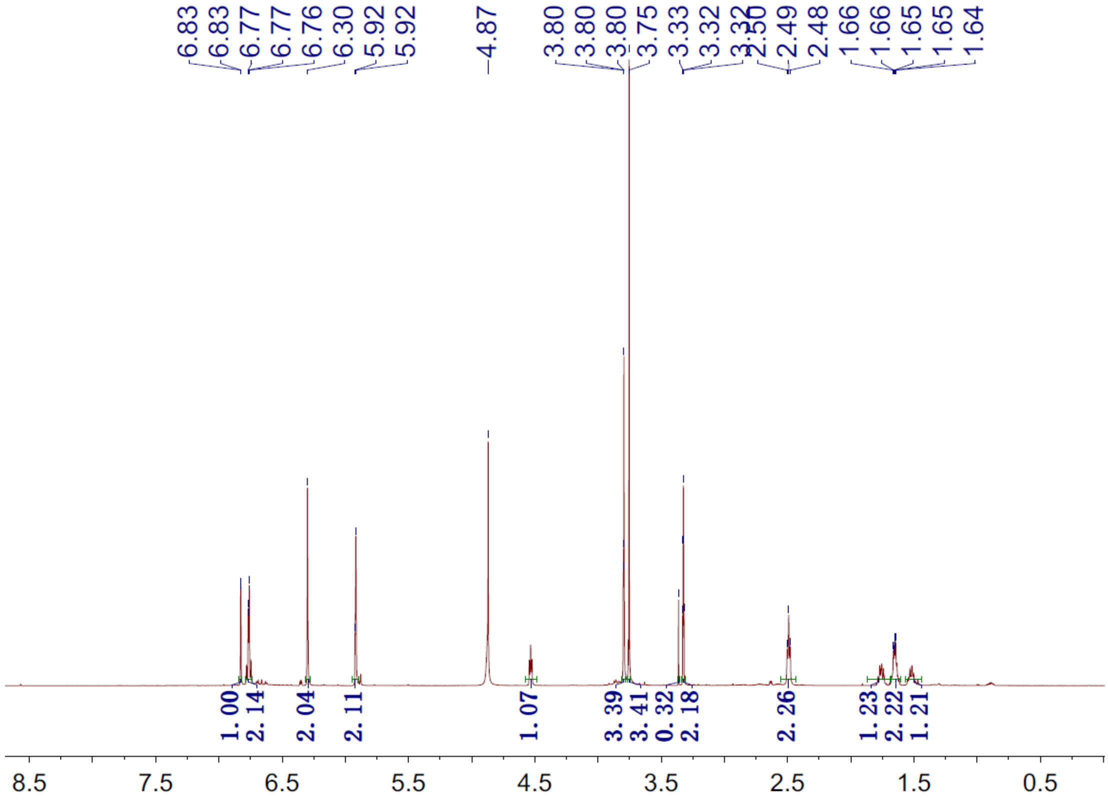

Figure S29. ^{1}H NMR spectrum of **3** in MeOH-d_4

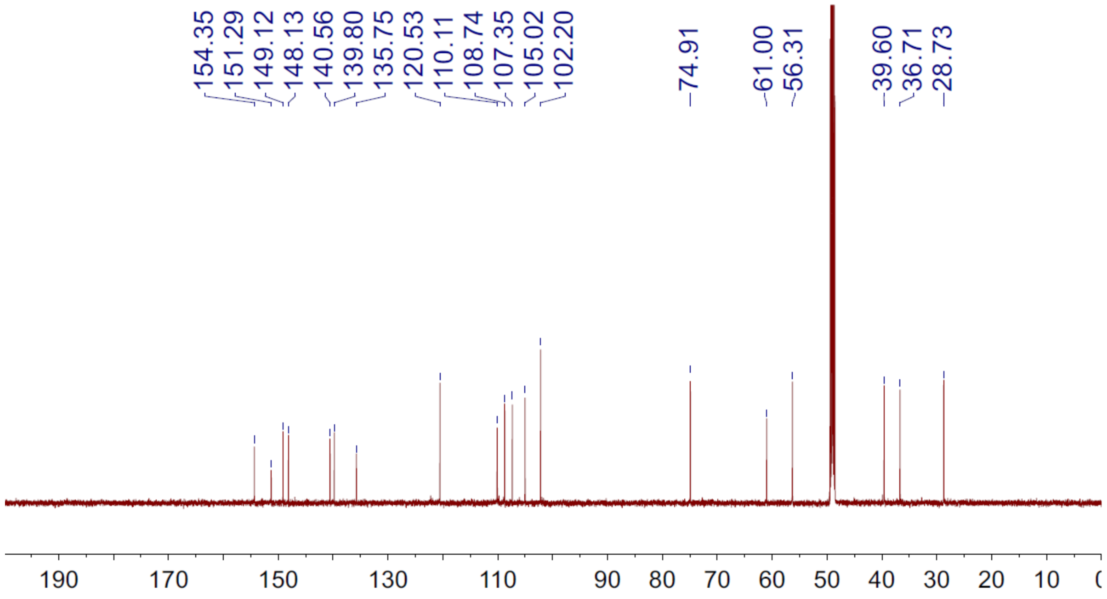

Figure S30. ^{13}C NMR spectrum of **3** in MeOH-d_4

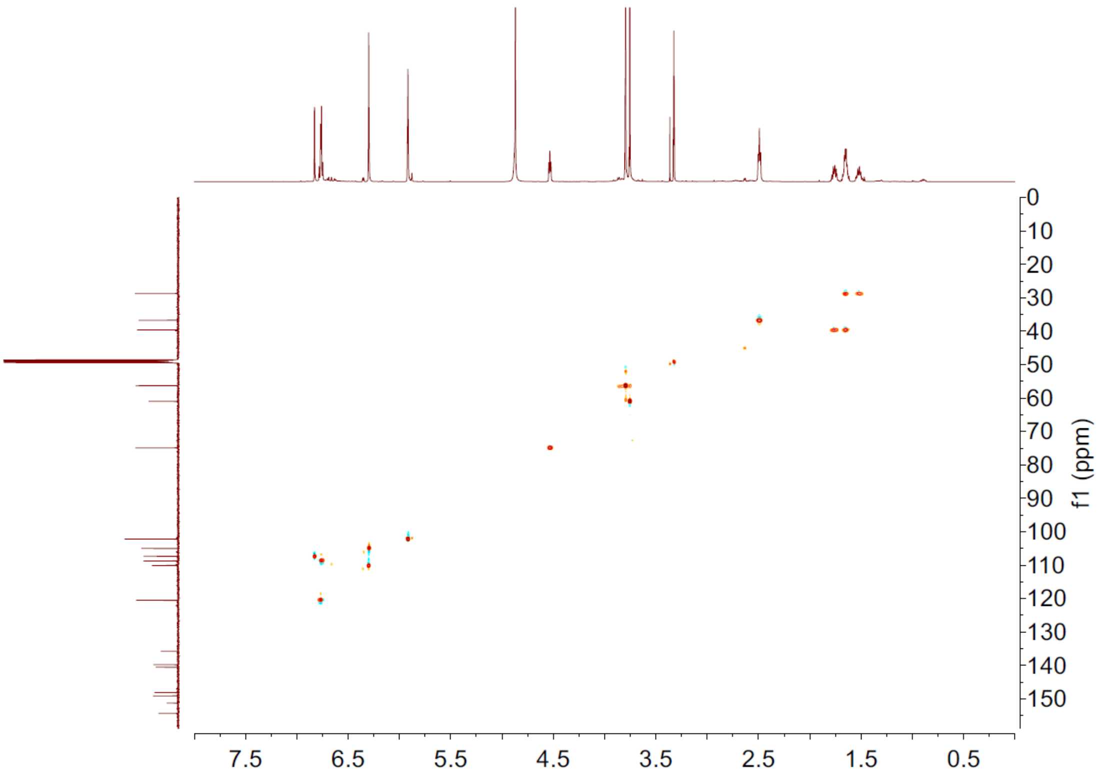

Figure S31. HSQC spectrum of **3** in MeOH-*d*$_4$

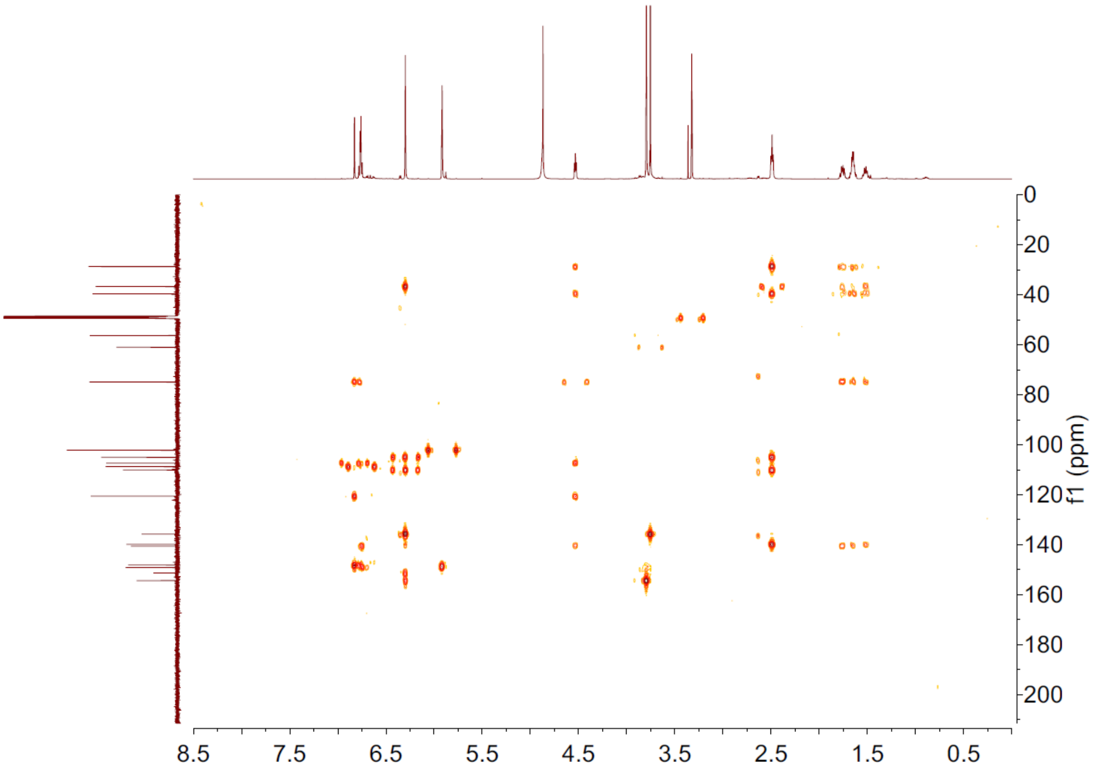

Figure S32. HMBC spectrum of **3** in MeOH-*d*$_4$

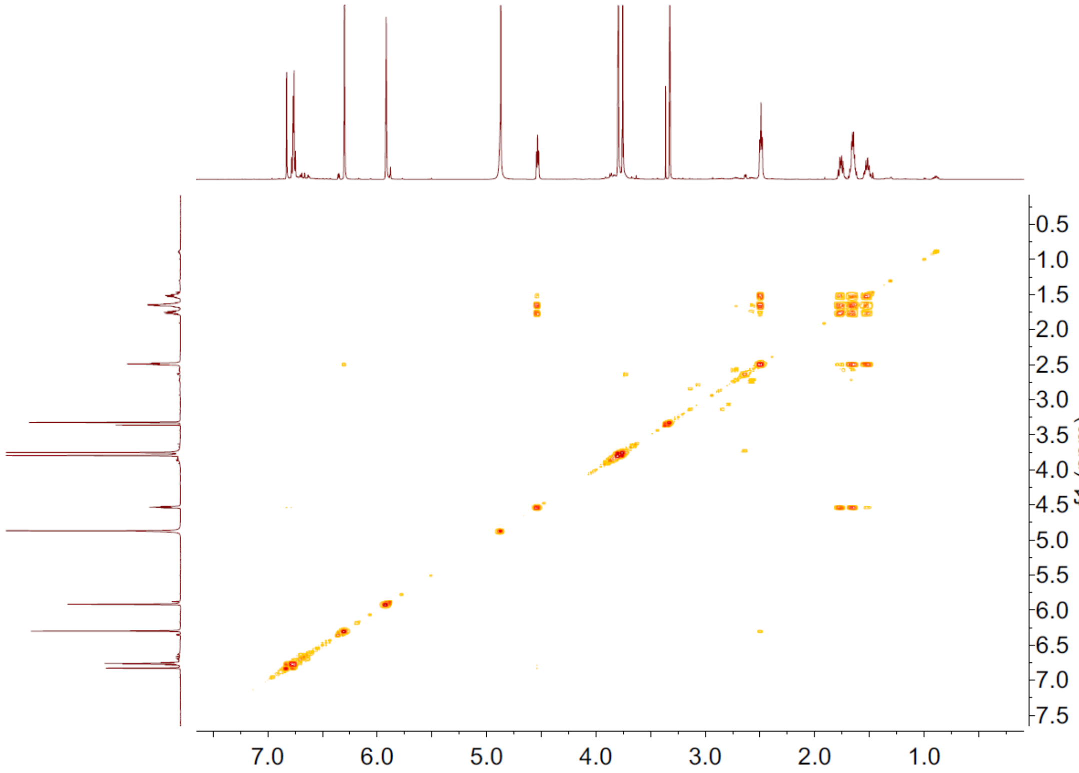

Figure S33. ^{1}H-^{1}H COSY spectrum of **3** in MeOH-d_4

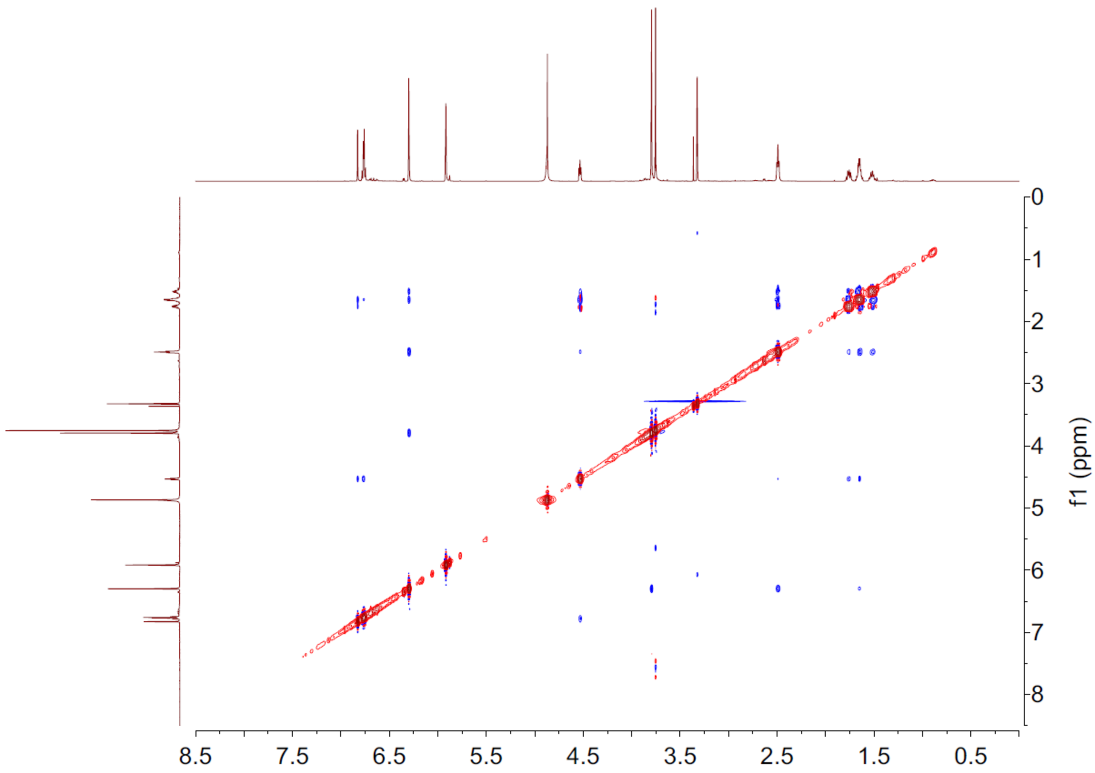

Figure S34. ROESY spectrum of **3** in MeOH-d_4

Qualitative Analysis Report

**Data Filename**	fzh-23.d	**Sample Name**	fzh-23
**Sample Type**	Sample	**Position**	P1-A6
**Instrument Name**	Instrument 1	**User Name**	
**Acq Method**	s-.m	**Acquired Time**	4/15/2022 4:27:57 PM
**IRM Calibration Status**	Success	**DA Method**	PCDL.m
**Comment**			

**Sample Group**		**Info.**
**Acquisition SW Version**	6200 series TOF/6500 series Q-TOF B.05.01 (B5125.2)	

User Spectra

Fragmentor Voltage	Collision Energy	Ionization Mode
135	0	ESI

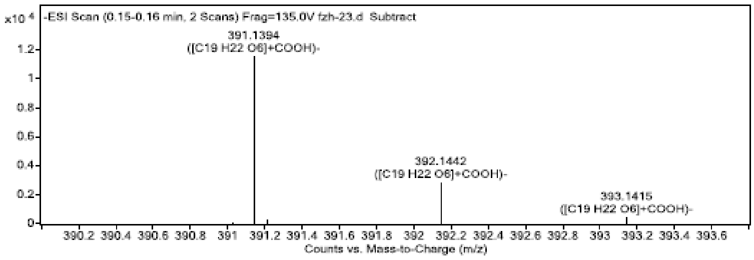

Peak List

m/z	z	Abund	Formula	Ion
89.0243	1	4327.62		
112.9856	1	7537.66		
154.9733	1	3112.79		
174.9557	1	4323.54		
248.9598	1	4478.24		
345.1334	1	7207.59		
391.1394	1	11556.99	C19 H22 O6	(M+COOH)-
392.1442	1	2893.67	C19 H22 O6	(M+COOH)-
681.2953	1	5033.48		
729.2553	1	2400.01		

Formula Calculator Element Limits

Element	Min	Max
C	3	60
H	0	120
O	0	30

Formula Calculator Results

Formula	CalculatedMass	CalculatedMz	Mz	Diff. (mDa)	Diff. (ppm)	DBE
C19 H22 O6	346.1416	391.1398	391.1394	0.40	1.02	9.0000

--- End Of Report ---

Figure S35. HRESIMS spectrum of **3**

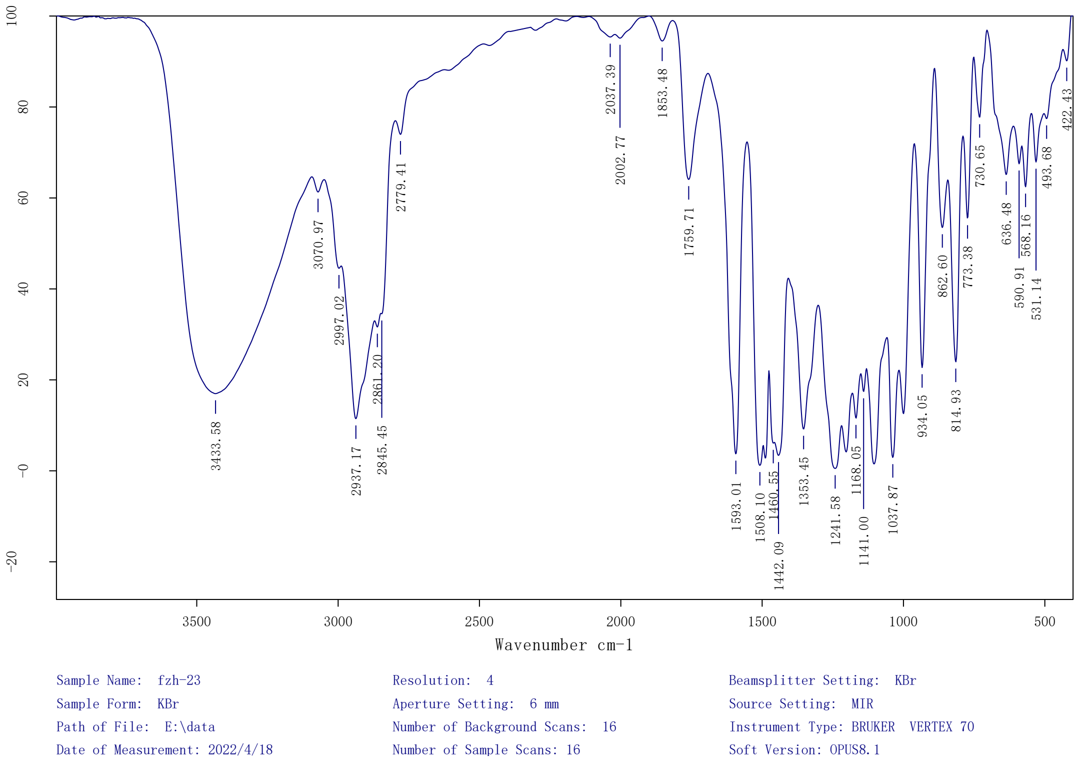

Figure S36. IR spectrum of **3**

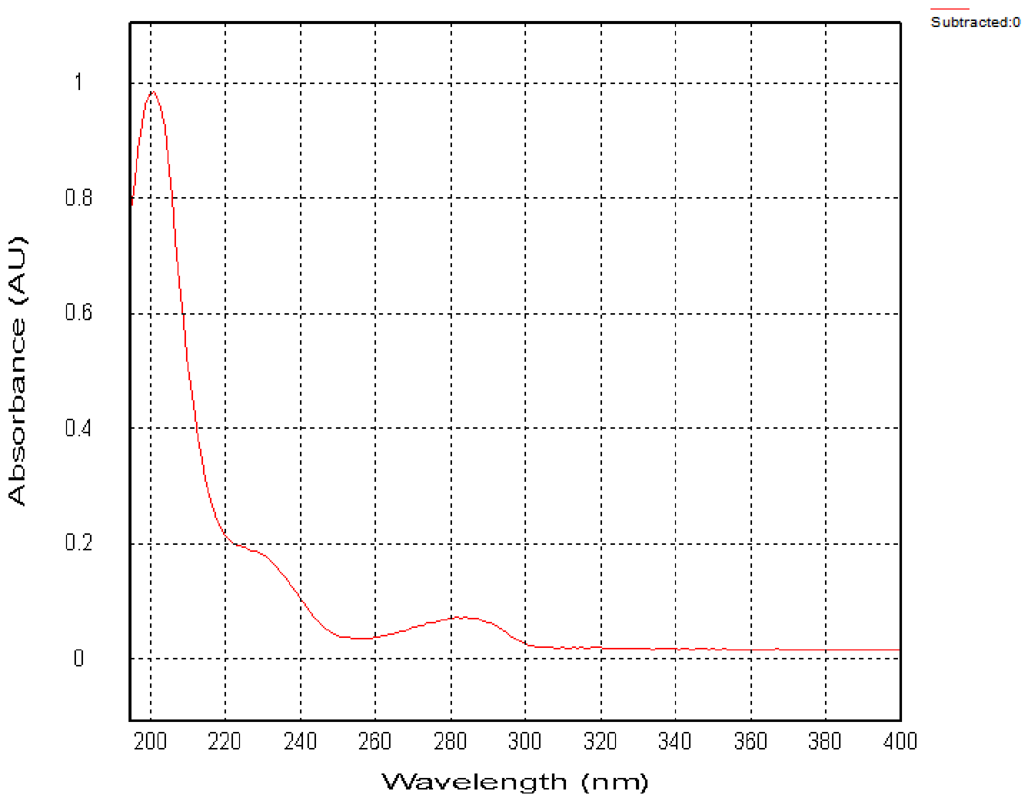

Figure S37. UV spectrum of **3**

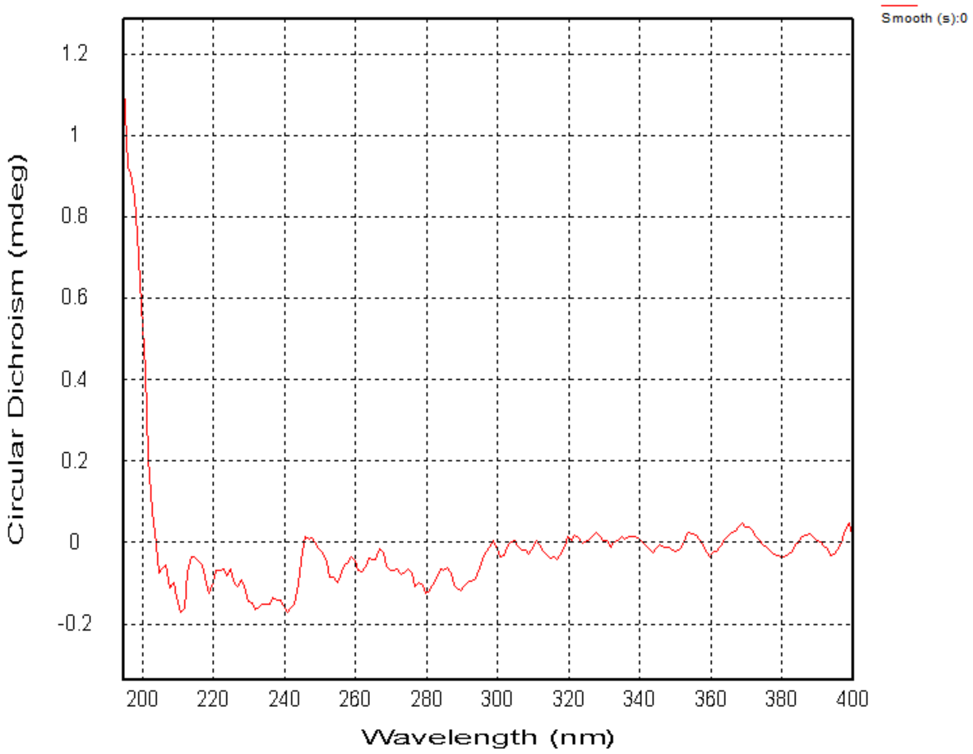

Figure S38. Experimental ECD curves of compounds **3**
